# Microscopic Response Mechanism of Epsilon-Negative and Epsilon-Near-Zero Metacomposites

**DOI:** 10.34133/research.0556

**Published:** 2025-02-01

**Authors:** Yunlei Zhou, Yanan Wang, Shicheng Qiu, Wei Zhao, Shaolei Wang, Hong Bao, Yunpeng Qu, Zhen Wen

**Affiliations:** ^1^ Hangzhou Institute of Technology, Xidian University, Hangzhou 311231, China.; ^2^ State Key Laboratory of Intelligent Manufacturing Equipment and Technology, Huazhong University of Science and Technology, Wuhan 430074, China.; ^3^ Department of Bioengineering, University of California, Los Angeles, Los Angeles, CA 90095, USA.; ^4^ College of Physics, Guizhou University, Guiyang 550025, China.; ^5^ Institute of Functional Nano and Soft Materials (FUNSOM), Jiangsu Key Laboratory for Carbon-Based Functional Materials & Devices, Soochow University, Suzhou 215123, China.

## Abstract

Metals have traditionally served as the primary functional phase in the development of metamaterials exhibiting epsilon-near-zero (ENZ) and epsilon-negative (EN) responses, albeit with persisting ambiguities regarding their response mechanisms. This paper presents the tunable ENZ (*ε*′ ~ 0) and EN (*ε*′ < 0) parameters at the 20-MHz to 1-GHz region based on Cu/CaCu_3_Ti_4_O_12_ (Cu/CCTO) metacomposites. By means of first-principles calculations and multi-physics simulations, the underlying mechanisms governing ENZ and EN responses are unveiled. The intricate pathways through which metacomposites achieve 2 dielectric response mechanisms are delineated: At low Cu content, a weak EN response (|*ε*′| < 200) was excited by electric dipole resonance, accompanied by ENZ effect; conversely, at high Cu content, due to the increase in effective electron concentration, plasmonic oscillation behavior occurs in the constructed 3-dimensional Cu network, resulting in strong EN response (|*ε*′| ~ 1,000) in the radio frequency band. These phenomena are explicated through 2 distinct Cu/CCTO models: Cu in an isolated state and a connected network state. This study not only comprehensively elucidates the 2 EN response mechanisms achieved by typical metacomposites with metals as functional phases but also delves into their associated electromagnetic shielding and thermal properties, providing a theoretical basis for their practical applications.

## Introduction

Since epsilon-near-zero (ENZ, *ε′* ~ 0) and epsilon-negative (EN, *ε′* < 0) responses have been experimentally achieved by fabricating metamaterials with periodic array structure, the extraordinary electromagnetic (EM) parameters widely trigger several brand-new physical effects [1–3]. These phenomena encompass negative refraction, reversed doppler effects, and the inverse Cherenkov effect [3–5]. This innovative design paradigm has not only led to breakthroughs in technologies such as invisibility cloaks, perfect lenses, EM shielding and absorption, zero refractive index mediums, and meta-capacitors but also revolutionized modern information and communication technology, electronic components, and other domains [4–6]. According to the different types of microstructures that achieve extraordinary EM parameters, EM metamaterials can be mainly divided into 2 categories: array metamaterials, characterized by functional phases periodically distributed on 2-dimensional (2D), 3D, and other scales, where their EM responses are designed and controlled by changing geometric configurations, array arrangements, etc.; the second type is random metamaterials, often referred to as metacomposites, because they mainly achieve extraordinary response by constructing multiphase composites such as binary and ternary materials [7–9]. Within these metacomposites, functional phases are typically scattered randomly within the matrix phase, and the manipulation of their morphology, dimensions, and distribution state plays a pivotal role in attaining desired performance benchmarks [10–12].

Metals have emerged as the predominant functional phase for metacomposite construction owing to their inherent plasmonic oscillation characteristics within the optical frequency range [13,14]. Notably, in the radio frequency (RF) band where the frequency is much lower than the optical frequency, the *ε′* of single-phase metals is a negative number on the order of 10^8^, while imaginary permittivity (*ε*″) takes a comparatively larger positive value [15]. Consequently, the RF dielectric constant of conductors like metals is an imaginary number. In response to this, metacomposites have been conceptualized as a viable solution. By integrating a conductor/insulator random composite structure, the effective carrier concentration of single-phase metals is significantly reduced, and the plasmonic oscillation frequency can be lowered to the RF band [16]. Then, the achieved *ε′* transitions from a theoretically meaningless imaginary quantity to a tangible value, effectively managing energy losses [17]. For instance, Fan and colleagues [18] constructed a randomly distributed Ni particle network in BaTiO_3_ matrix through in situ reduction process, and achieved EN and ENZ response by regulating the metal structure. Using a similar method, they constructed EN metacomposites with BaTiO_3_ ceramic matrix, varying the metal type and network structure to explore high-frequency magnetic responses and thermal properties [19]. Beyond ceramics, polymers can serve a similar role as insulating matrices. For example, Wu and colleagues [20] loaded carbon-modified Ni particles with different contents on polyvinyl alcohol as a flexible matrix, and achieved broadband ENZ response through magnetic field driving. It is worth noting that in recent years, research has shown that carbon nanomaterials can also serve as effective functional phases to construct metacomposites, similar to metals, because the 3D carbon network structure can achieve a metal-like plasmonic oscillation effect [21,22]. Graphene, carbon nanotubes, and other carbon materials can also achieve properties similar to metals and greatly expand the connotation of metacomposites, due to their controllable geometric configurations and moderate electrical performance [23,24]. Overall, metals and carbon materials as functional phases of metacomposites have their own advantages and disadvantages in terms of frequency band, dispersion, and loss of EN response, which urgently requires further comprehensive evaluation and research. However, current research on metacomposites is mostly limited to material design and performance control, resulting in a lack of clear elucidation of the micromechanisms of EN and ENZ responses, which undoubtedly hinders the development of applications in the field of metacomposites. Urgently, attention should also be paid to the heat dissipation issues related to the losses of EN and ENZ media in practical applications.

Therefore, we chose typical Cu medium as the functional phase and insulated copper calcium titanate [CaCu_3_Ti_4_O_12_ (CCTO)] ceramics as the matrix phase. Based on the percolation system, we constructed a binary composite and successfully achieved EN and ENZ response at the RF region. The former was chosen for its excellent conductivity, thermal conductivity, and chemical stability, commonly employed as a fundamental unit in metamaterial applications, while the latter was selected for its special interlayer capacitance structure that can effectively limit the conduction loss of metal networks while maintaining high-temperature structural stability [24–26]. Unlike previous works where CCTO was simply used as an insulating matrix, Cu/CCTO metacomposites enrich the regulation scope of EN response by introducing the dielectric resonance mechanism of CCTO. We used computed tomography (CT) technology to characterize the 3D composite structure to assist in explaining the intrinsic correlation between structure and properties of Cu/CCTO composites. Furthermore, we used first-principles calculations and EM simulations to investigate the micromechanisms of EN and ENZ response.

## Results and Discussion

### Structural and compositional characterization

**Fig. 1.**
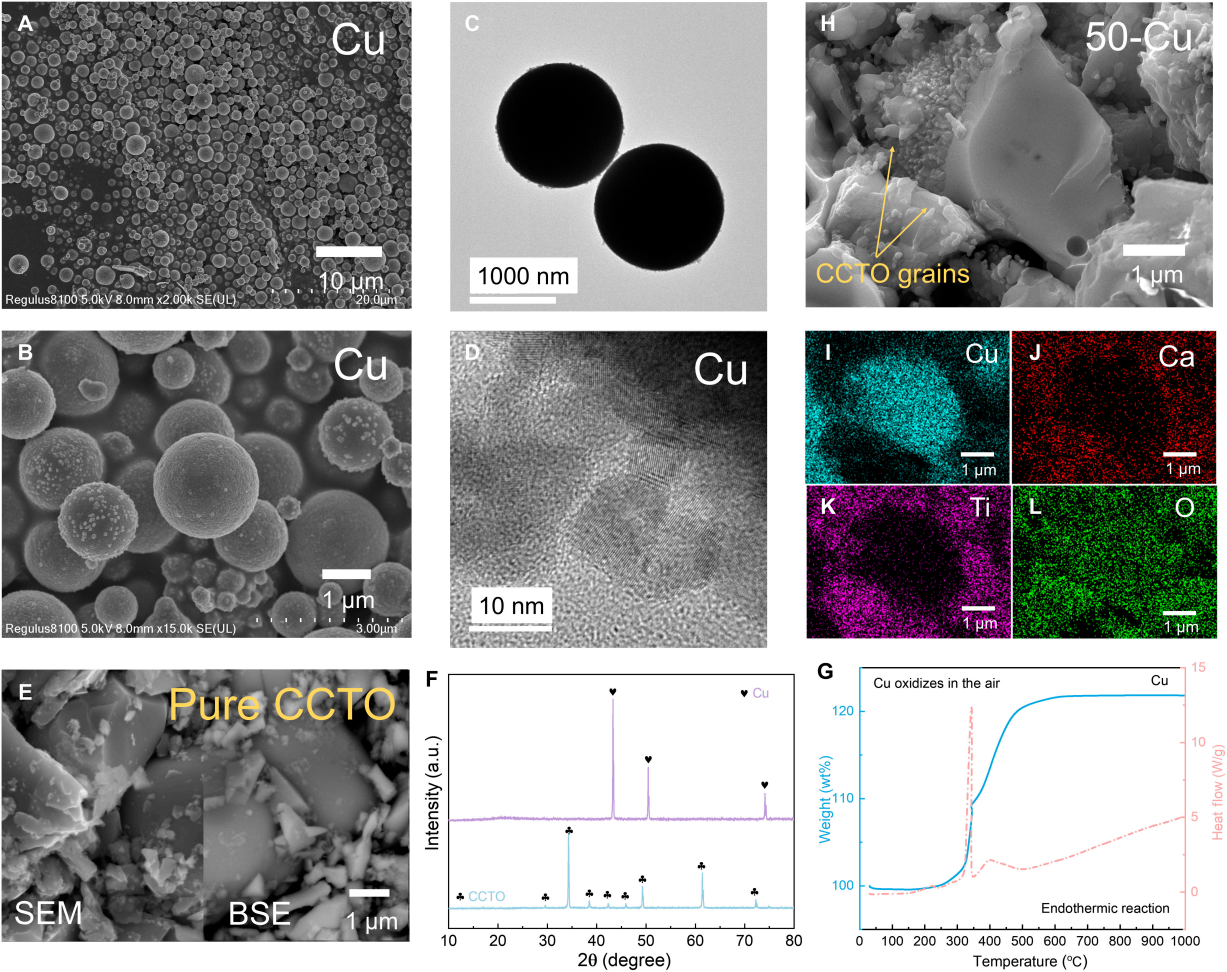
(A to D) FESEM and TEM images of Cu powders. (E) FESEM image and corresponding BSE image of CCTO raw powders. (F) XRD patterns of Cu and CCTO powders. (G) TG-DSC curves of Cu powders with heating rate of 10 °C/min. (H to L) FESEM and EDS images of Cu/CCTO composites with Cu content of 50 wt %.

The subsequent sections present an in-depth analysis of the microstructural evolutions observed in Cu/CCTO composites, triggering a conventional electrical percolation phenomenon. First, the morphology of our carefully selected 0D spherical Cu particles as the metal functional phase was demonstrated using field-emission scanning electron microscopy (FESEM) and transmission electron microscopy (TEM) techniques, as shown in Fig. 1A to D and Figs. S1A to H and S2A to P. Evidently, these Cu particles exhibit a relatively consistent size, approximately 500 nm in diameter, and possess favorable formability characteristics, facilitating the establishment of a robust 3D network within the matrix. Similarly, SEM and backscattered electron (BSE) images were used to demonstrate the CCTO powder samples synthesized by solid-state reaction method (Fig. 1E and Fig. S3A to J). Variances in the sizes of CCTO particles were noted, hinting at potential challenges in achieving homogeneous dispersion when mixed with Cu powder, a matter we intend to reevaluate after the successful sintering process. Prior to the fabrication of Cu/CCTO composites, x-ray diffraction (XRD) patterns in Fig. 1F affirm the component purity of the Cu and CCTO raw materials, with no discernible impurities observed in the synthesized CCTO powder. In order to ensure the stability of Cu particles during the preparation process and heat dissipation conditions, we analyzed their thermal reaction at high temperatures using TG-DSC (thermogravimetric analysis–differential scanning calorimetry) technology, as shown in Fig. 1G. It is demonstrated that Cu in air undergoes endothermic reactions with oxygen and increases in weight as the temperature rises, especially between 300 and 500 °C, where the reaction is most intense. After reaching over 600 °C, the mass increases to around 122 wt % and reaches stability, because the copper oxide formed on the surface of the particles protects the Cu particles and gives them better thermal stability.

Furthermore, we incorporated Cu particles into the CCTO matrix with a varying content gradient, and characterized the internal cross-sectional morphology of the Cu/CCTO composites using FESEM, BSE, and EDS (energy-dispersive spectroscopy), as shown in Fig. 1H to L and Figs. S4 to S10. As the Cu content increases, a gradual formation of a 3D network, engineered by Cu as the functional phase, emerged. As shown in Movie S1, we obtained the microstructure inside the Cu/CCTO composites through CT technology. It can be seen that the Cu functional phase and CCTO matrix form a randomly and uniformly distributed composite structure without obvious macroscopic defects. While precise quantification of the percolation threshold within the morphology map remains challenging, accurate information on this percolation behavior can be provided by testing its subsequent electrical properties. Given the dual presence of Cu present in the functional phase and the matrix phase of Cu/CCTO composites, it becomes imperative to ascertain the existence of a conductive Cu network by analyzing the distribution of other elements in the BSE images and EDS spectra. Noteworthy regions exhibiting enhanced conductivity, represented by white areas in the BSE images, signify the presence of randomly dispersed copper elemental clusters and networks. Furthermore, the XRD pattern of the composites (Fig. S4A) clearly reveals the increase in Cu elemental content and the stable existence of CCTO phase.

### Electrical percolation

**Fig. 2.**
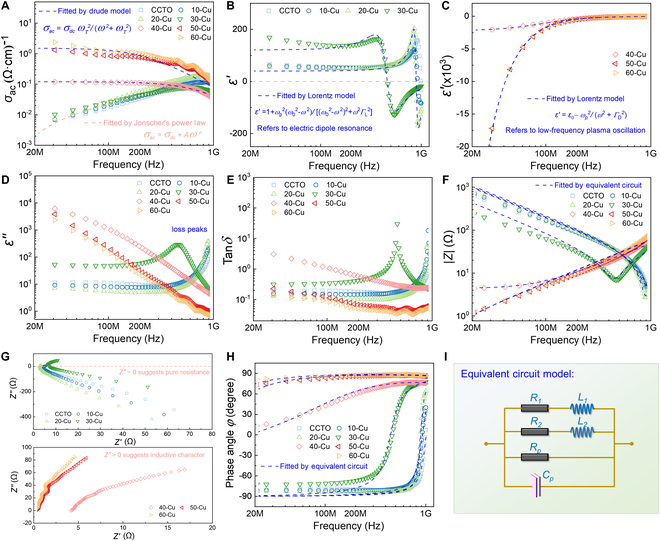
Frequency dependences of AC conductivity (A), complex permittivity (B to D), loss tangent angle (E), impedance modulus (F), and phase angle (H) for Cu/CCTO composites. Nyquist plots (G) for Cu/CCTO composites. Schematic of equivalent circuit used for analysis (I), which presents as dashed lines of fitting results in (F) and (H).

We qualitatively analyzed the percolation evolution of Cu functional phase in Cu/CCTO composites through structural morphology characterization. Our characterization of its electrical conductivity indicates the electrical percolation behavior of the composites. As illustrated in Fig. 2A, the variation of alternating current (AC) conductivity (*σ_ac_*) with frequency for Cu/CCTO composites is depicted. It clearly shows 2 different trends of *σ_ac_* variation, a significant increase with increasing test frequency at low Cu content, contrast by a gradual decrease at high Cu content with a more pronounced trend at high frequencies. This observation suggests the existence of dual conductivity mechanisms within the composites. In the former scenario, Cu/CCTO composites can be regarded as an amorphous semiconductor, where electrons in the localized state are bound to a local region in space. Electrons need the assistance of phonons to transition from one localized state to another and can undergo hopping conductivity, which can be described by Jonscher’s power law: [27,28]

$$\sigma_{\mathrm{ac}}=\sigma_{\mathrm{dc}}+A{\left(2\pi f\right)}^{n}$$ (1)

where *σ_dc_* represents the direct current conductivity, *f* denotes the test frequency, *A* is the pre-exponential factor, and *n* is the fractional exponent (0 < *n* < 1). Note that this jump only occurs when the state of the electron jumps and the position of the electron in real space do not change. Hopping conductivity allows insulating or semiconducting dielectric materials to achieve better conductivity at high frequencies, but correspondingly, it can damage the polarization ability of the dielectric material, thereby affecting its ability to store charges. For 40-Cu, 50-Cu, and 60-Cu samples, the decreasing trend of AC conductivity corresponds to the skin effect widely present in metal-like conductivity. Specifically, when there is AC or AC EM field in the conductor, the current distribution inside the conductor is uneven, and the current is concentrated in the “skin” part of the conductor; in other words, the current is concentrated in the thin layer on the surface of the conductor. This phenomenon signifies that the current density increases as one moves closer to the surface of the conductor, which can be described using the Drude model [29,30]:

$$\sigma_{\mathrm{ac}}=\frac{\sigma_{\mathrm{dc}}\omega_{\tau }^{2}}{{\left(2\pi f\right)}^{2}+\omega_{\tau }^{2}}$$ (2)

$$\sigma_{\mathrm{dc}}=\frac{Ne^{2}\tau }{m}=\frac{\omega_{p}^{2}\tau }{4\pi }$$ (3)

Here, *ω_τ_* (*ω_τ_* = 1/*τ*) is the relaxation rate, *ω_p_* (*ω_p_* = 2π*f*_p_) is the plasma frequency, *e* is the electron charge (1.6 × 10^−19^ C), *N* is the Avogadro constant, and *m* is the electron mass. At this point, Cu/CCTO composites can be considered as good conductors, and thus, the magnetic field generated by high-frequency current in the conductor induces the maximum electromotive force in the central area of the wire. Due to the induced electromotive force generating induced current in a closed circuit, the maximum induced current occurs at the center of the wire. The induced current consistently decreases in the direction of the original current, and it forces the current to be limited to the outer surface of the wire. This effect primarily arises from the generation of a vortex electric field within the conductor due to the altering EM field, counteracting the original current. The skin depth (*δ*) of this effect can also be quantitatively described [31]:

$$\delta =\sqrt{\frac{2}{2\mathrm{\pi f\mu}\sigma_{\mathrm{dc}}}}$$ (4)

where *μ* indicates static permeability. Evidently, this elucidates why the skin effect becomes more pronounced at high frequencies. The dispersion behavior at high frequencies is due to the heterogeneity effect of the Cu/CCTO composites. Cu and CCTO matrix form a large number of irregular interfaces, which exacerbates the dispersion of conductivity when the skin effect occurs at high frequencies. Although the Cu/CCTO composites we constructed are a heterogeneous dielectric material, its macroscopic electrical properties can still be accurately quantitatively described. By distinguishing these 2 different conductivity mechanisms, we have effectively clarified the electrical percolation behavior of Cu/CCTO composites, with a percolation threshold between 30 and 40 wt %.

### EN and ENZ responses

The presence of electrical percolation behavior indicates that the carrier transport characteristics inside Cu/CCTO composites will inevitably change with variations in the Cu functional phase, thereby affecting their high-frequency dielectric properties. Figure 2B and C shows the frequency spectrum of the real permittivity (*ε′*) of composites across the frequency range of 20 MHz to 1 GHz. Corresponding to the percolation behavior revealed by the conductivity spectrum, Cu/CCTO composites achieved 2 different EN responses. Specifically, in CCTO, 10-Cu, 20-Cu, and 30-Cu samples, *ε′* changes from positive to negative as the frequency of the applied electric field increases, which can be referred to as a typical EN response of dielectric resonance type. Notably, these samples manifest an ENZ state near the resonance frequency, achieving a narrow EN bandwidth. This behavior is well described by the Lorentz model [32,33]:

$$\varepsilon^{\prime }=1+\frac{\omega_{p}^{2}\left(\omega_{0}^{2}-\omega^{2}\right)}{{\left(\omega_{0}^{2}-\omega^{2}\right)}^{2}+\omega^{2}\Gamma_{L}^{2}}$$ (5)

where *ω* (*ω* = 2π*f*) is the frequency of the electric field, *ω*_0_ is the resonance frequency, and Γ*_L_* is the damping constant. Upon exposure to an electric field, the internal electric dipoles within the Cu/CCTO composites will be polarized. This polarization becomes notably pronounced when the electric field frequency approaches the natural resonance frequency of the material, resulting in an enhanced response of the material at this frequency. Generally, the weakly EN response was defined as the absolute value of real permittivity *ε′* smaller than 1,000 (0 < |*ε′*| < 1,000).

In the case of composites where a 3D conductive Cu network has been established within the CCTO matrix after percolation, their EN response can span a broader frequency spectrum (20 MHz to 1 GHz), with the absolute value of *ε′* decreasing as the applied electric field frequency increases. This particular EN response comes from the low-frequency plasmonic oscillation state generated by the excitation of a multitude of free electrons in the Cu network by an alternating electric field. When the oscillation frequency of delocalized electrons is lower than the frequency of the external electric field, EN response may be exhibited, as schematic in Fig. 5A to C. The Drude model explains this type of EN response [34,35]:

$$\varepsilon^{\prime }=\varepsilon_{\infty }-\frac{\omega_{p}^{2}}{\omega^{2}+\Gamma_{D}^{2}}$$ (6)

$$\omega_{p}=\sqrt{\frac{n_{\mathrm{eff}}e^{2}}{m_{\mathrm{eff}}\varepsilon_{0}}}$$ (7)

Here, *ε*_0_ is the vacuum permittivity (8.85 × 10^-12^ F/m), *ε*_∞_ represents the high-frequency limit permittivity, *Γ*_D_ is the damping constant, and *n*_eff_ and *m*_eff_ are the effective concentration and mass of free carriers, respectively. From Fig. 2C, it reveals that the fitting results align with the experimental data. It is worth noting that when the Cu content within the composites reaches 50 wt % or higher, the magnitude of *ε′* exhibits minimal variance, indicative of the saturation of the plasmonic state within the 3D Cu network. Subsequent increments in Cu content have little effect on improving the magnitude of *ε′*. According to the Drude model, for Cu/CCTO metacomposites, the main factor determining the frequency band and magnitude of EN response is *ω_p_*, which is closely related to the carrier concentration inside the material.

In summary, the Drude type generally exhibits a stronger EN response than the Lorentz type, with an *ε′* order of magnitude higher, reaching 10^3^ to 10^4^. Correspondingly, the former will experience higher energy loss in practical applications, which is usually evaluated by the imaginary permittivity (*ε*″) and loss tangent angle (tan*δ* = |*ε*″/*ε′*|), as shown in Fig. 2D and E. *ε*″ is directly related to the material’s ability to absorb EM waves. It describes the energy loss caused by an electric field inside the material. This form of energy dissipation typically translates into the attenuation of EM waves as they traverse through materials. Therefore, the different trends in *ε*″ alternations before and after percolation in Cu/CCTO composites also imply different internal loss mechanisms, which can be explained by interface polarization loss (*ε*_p_″), dipole loss (*ε*_D_″), and conduction loss (*ε*_C_″) [36]:

$$\varepsilon^{\prime \prime }=\varepsilon_{P}^{\prime \prime }+\varepsilon_{D}^{\prime \prime }+\varepsilon_{C}^{\prime \prime }$$ (8)

where *ε*_p_″ plays a major role in the low-frequency range (below 1 MHz), while *ε*_D_″ and *ε*_C_″ make a major contribution in our test frequency region. Obviously, there is a loss peak in the composites near the dielectric resonance frequencies. The peaks in dielectric loss signify the maximal electrical energy dissipation of Cu/CCTO composites at specific frequencies. When these composites shift from positive dielectric properties to EN properties, they necessitate energy consumption to drive electric dipole resonance. This concept is intuitive since resonant states require more energy than polarized states. In the 3D conductive copper network constructed by further increasing the content of Cu functional phases in composites, *ε*_C_″ plays a dominant role in achieving EN response, which corresponds to the skin effect analyzed earlier. Both *ε*″ and tan*δ* exhibit a gradual descent as frequency escalates, corresponding to a gradual decrease in transmission loss. This is because the plasmonic state has been greatly weakened at high frequencies, and the amplitude of the EN response has decreased. It is worth noting that the Cu/CCTO composites we obtained has higher losses compared to traditional positive-*k* dielectric materials. This is because EN materials have different application scenarios, such as lossy EM wave absorbing materials, EM shielding materials, and broadband ENZ devices.

### Equivalent circuit analysis

Through electrical and dielectric analysis of the Cu/CCTO composites we constructed, we have elaborated on their percolation effect and EN mechanism. Subsequently, we employ equivalent circuit analysis to study the impedance characteristics of the material in order to evaluate the application of EN materials in new electronic components and other fields, as shown in Fig. 2F to I. The impedance modulus (|*Z*|) of Cu/CCTO composites exhibits 2 frequency-dependent trends. The decline of |*Z*| with frequency can be attributed to the diminishing capacitive nature, correlating with the hopping conductivity behavior exhibited by the medium. Conversely, it is noteworthy that the |*Z*| of Cu/CCTO composites increases with frequency at high-frequency range, due to the increase in its inductance characteristics. For composites with higher Cu content, this trend is more significant, so we used an equivalent circuit that includes capacitor (*C_p_*), resistors (*R*_1_, *R*_2_, and *R_p_*), and inductors (*L*_1_ and *L*_2_) to fit the impedance spectrum, as shown in Fig. 2I. These resistors and capacitors mainly come from the internal barrier layer capacitor (IBLC) structure of the CCTO matrix, while inductive elements mainly come from the 3D Cu network. The relationship between the signs of reactance (*Z*″) and dielectric properties can be more clearly established according to the following formula [37]:

$$\varepsilon^{\prime }=\frac{-Z^{\prime \prime }}{2\pi fC_{0}\left(Z^{\prime 2}+Z^{\prime \prime 2}\right)}$$ (9)

where *C_0_* represents the vacuum capacitance and *Z′* is resistance. Here, the *Z*″ value of positive-*k* materials is negative, indicating that they are dominated by capacitive characteristics. Correspondingly, the reactance value of EN media is positive, indicating that they have inductive characteristics. This is because *Z*″ is composed of inductive reactance (*Z_L_*) and capacitive reactance (*Z_C_*): *Z*″ *= Z_L_* − *Z_C_*. As shown in Fig. 2G, before percolation, the *Z*″ value of Cu/CCTO composites changes from negative to positive with the increase of frequency, and its transition frequency corresponds exactly to the ENZ frequency of the material, at which frequency the dielectric medium is in pure resistive state. As the Cu content increases, the *Z*″ value of the composites remains positive throughout the entire testing frequency, indicating that the EN response medium has inductance, which can be verified from the phase relationship diagram shown in Fig. 2H. The positive value of phase angle (φ) indicates that voltage leads current through an inductor. In addition, when the phase value is zero, it corresponds to the ENZ point of Cu/CCTO composites. At this time, the dielectric medium is purely resistive, and the voltage and current phases are synchronized. Through the method of equivalent circuit analysis, we have successfully elucidated the intrinsic inductance of EN materials, which is closely related to the conductive Cu network constructed inside Cu/CCTO composites. In other words, the randomly distributed Cu functional phases can be equivalent to a large number of micro-inductors under the action of high-frequency electric fields. In summary, dielectric materials with EN and ENZ responses are expected to play an important role in the design of new metacapacitors, wireless wound inductors, sensors for EM wave controlled, and other fields [38–40].

### First-principle calculations and EM simulations

**Fig. 3.**
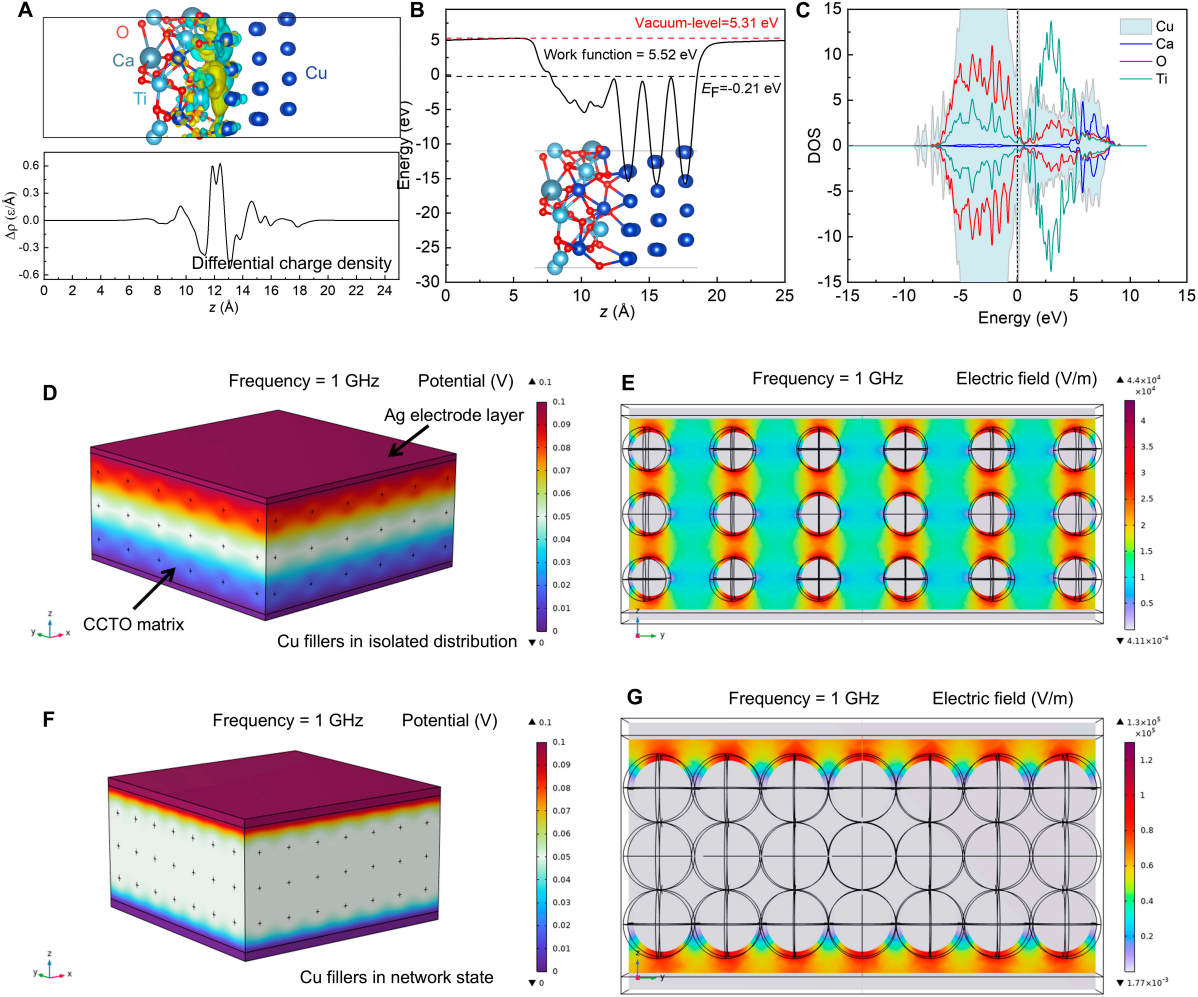
The first-principles calculation results of Cu@CaTi_4_(CuO_4_)_3_ heterostructures. (A) Differential charge density of Cu@CaTi_4_(CuO_4_)_3_. The yellow and cyan regions indicate an increase and decrease in electron density, respectively. (B) Work function and Fermi level. (C) DOS of Cu@CaTi_4_(CuO_4_)_3_ heterostructures. The EM simulation results of Cu/CCTO composites with isolation distribution state (D and E) and network state of Cu fillers (F and G).

In our investigation of typical binary metal/ceramic composites, we initiated by scrutinizing the charge transport properties at the Cu/CCTO interface utilizing first-principles calculations. The findings, as depicted in Fig. 3A to C, encompassed the differential charge density distribution, work function, and density of states (DOS) plots. Evidently, the crystal structure at the interface between Cu and CCTO has undergone rearrangement to attain a stable energy state, resulting in a large amount of charge transfer between the 2 and the formation of an electric dipoles, which is also the essential cause of dielectric resonance [41]. At the vicinity of Fermi level (−0.21 eV), the electronic states of Cu and Ti have orbital hybridization, suggesting the interaction between the orbitals of the transition metal atoms (Cu and Ti) in CaTi_4_(CuO_4_)_3_ and the Cu atoms of fillers. From the DOS graph, it can be seen that the Cu atoms belonging to the CCTO structure partially overlap with the Cu atoms belonging to the functional phase, but can be analyzed by corresponding Ti, Ca, and O atoms. This also explains why the pure CCTO sample we obtained has a dielectric resonance. Specifically, through the sintering process, CCTO forms an IBLC structure, which accumulates a large number of electric dipoles between its grains [42].

**Fig. 4.**
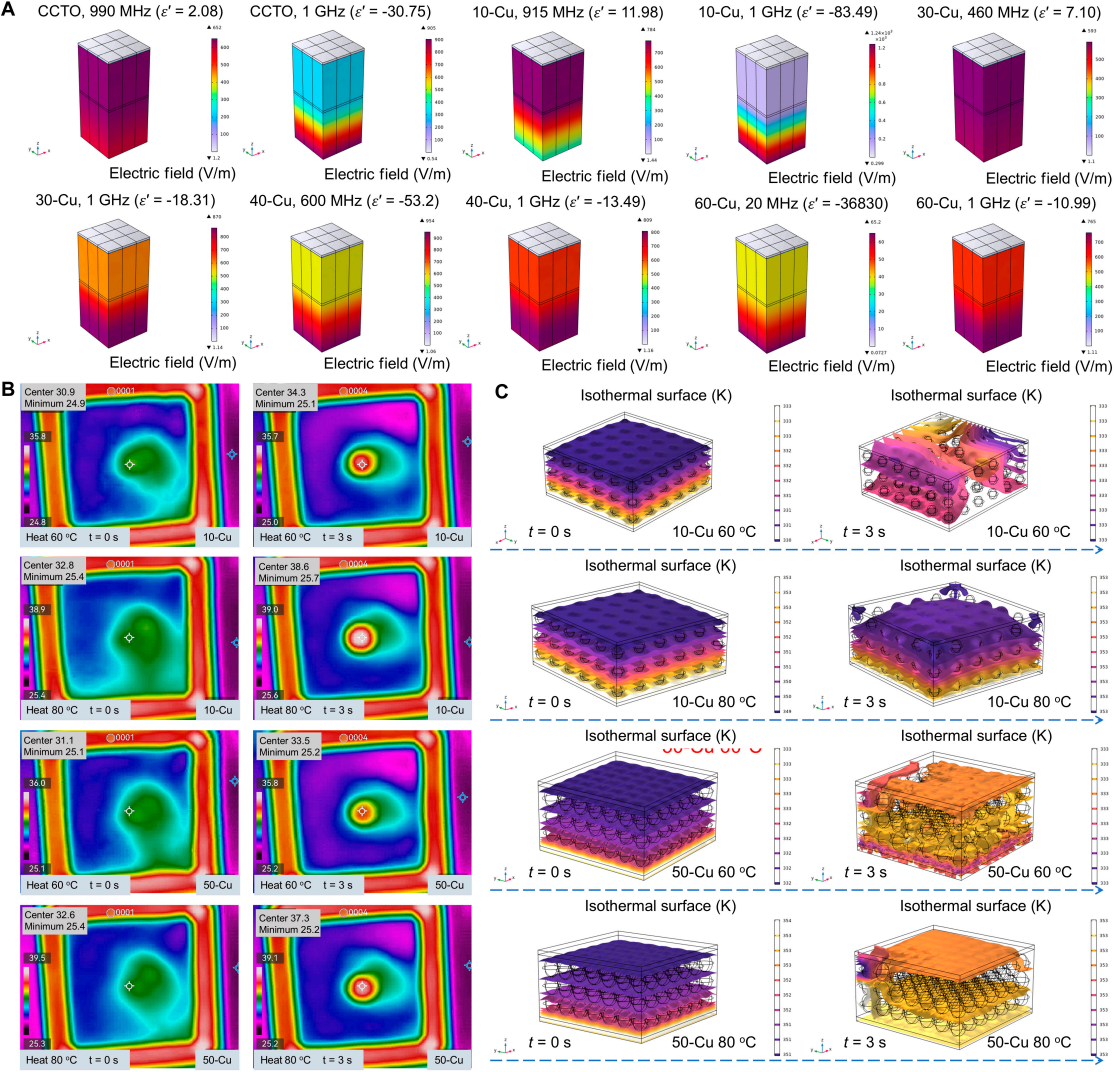
Electric field vector distribution at different EN and ENZ frequencies, highlighting variations in Cu content. (A) Temperature pattern at heating temperature of 60 and 80 °C for metacomposites with Cu content of 10 and 50 wt %. (B) Metacomposites with isolation distribution state of Cu fillers at 60 and 80 °C. (C) Metacomposites with network state of Cu fillers at 60 and 80 °C.

**Fig. 5.**
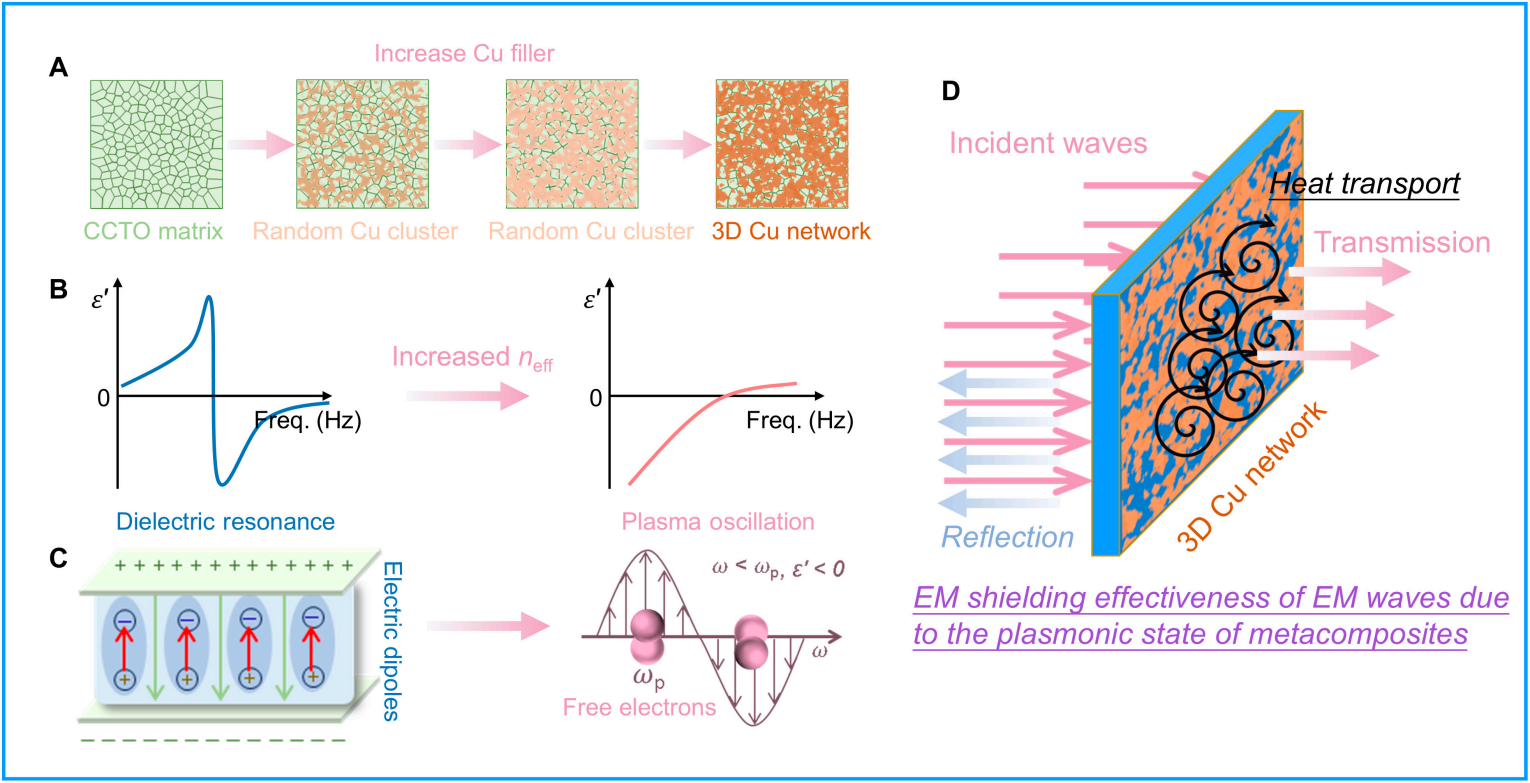
Schematic for EN response mechanism with increasing Cu fillers in CCTO matrix (A to C) and resultant EM shielding effect (D).

Zooming in on the macroscopic structure of Cu/CCTO, we adopted CCTO as a homogeneous matrix and Cu spherical particles as functional phases to establish 2 models in COMSOL software, namely, Cu isolated distribution and Cu network distribution, as shown in Fig. 3D to G. In the scenario where Cu particles are isolated in the CCTO matrix, owing to their commendable conductivity, the interior of Cu particles is in an equipotential state under the action of an external electric field, resulting in significant electric field redistribution between Cu particles, making adjacent particles equivalent to electric dipoles. Conversely, when the Cu particles are completely connected into a network, the entire model is in an equipotential state, and the surface material exhibits metal-like conductivity behavior [43]. The current is concentrated on the surface of the material, which is consistent with the skin effect we analyzed earlier. Furthermore, we assigned the measured dielectric data to the established model and revealed the effects of EN and ENZ materials on EM wave transmission in COMSOL software, as shown in Fig. 4A. Two critical observations surface from this analysis. First, as the amount of random conductive fillers increases, the percolation threshold is gradually reached, thereby affecting the EN response, as shown in Fig. 5A to C. Furthermore, EN materials have a more significant shielding effect on EM waves at low frequencies, but the effect weakens as the frequency increases, as schematic in Fig. 5D. This also proves that obtaining plasmonic oscillating state media is more superior in the MHz frequency band. Second, changes in the magnitude of EN values can significantly affect the transmission effect of EM waves through dielectric materials, indicating that the regulation of EN response is essential in applications such as EM wave manipulation at specific frequencies [44].

Zooming in on the macroscopic structure of Cu/CCTO, we adopted CCTO as a homogeneous matrix and Cu spherical particles as functional phases to establish 2 models in COMSOL software, namely, Cu isolated distribution and Cu network distribution, as shown in Fig. 3D to G. In the scenario where Cu particles are isolated in the CCTO matrix, owing to their commendable conductivity, the interior of Cu particles is in an equipotential state under the action of an external electric field, resulting in significant electric field redistribution between Cu particles, making adjacent particles equivalent to electric dipoles. Conversely, when the Cu particles are completely connected into a network, the entire model is in an equipotential state, and the surface material exhibits metal-like conductivity behavior [43]. The current is concentrated on the surface of the material, which is consistent with the skin effect we analyzed earlier. Furthermore, we assigned the measured dielectric data to the established model and revealed the effects of EN and ENZ materials on EM wave transmission in COMSOL software, as shown in Fig. 4A. Two critical observations surface from this analysis. First, as the amount of random conductive fillers increases, the percolation threshold is gradually reached, thereby affecting the EN response, as shown in Fig. 5A to C. Furthermore, EN materials have a more significant shielding effect on EM waves at low frequencies, but the effect weakens as the frequency increases, as schematic in Fig. 5D. This also proves that obtaining plasmonic oscillating state media is more superior in the MHz frequency band. Second, changes in the magnitude of EN values can significantly affect the transmission effect of EM waves through dielectric materials, indicating that the regulation of EN response is essential in applications such as EM wave manipulation at specific frequencies [44].

When EM waves are transmitted in EN media, losses are converted into heat, impacting the material’s operational stability [45]. To evaluate performance under varied excitation heat source temperatures, we employed infrared thermal imaging, as shown in Fig. 4B. In the case of the 10-Cu sample where Cu particles are isolated in the CCTO matrix, heat transmission induces vibration proximate to the equilibrium position of the CCTO lattice structure. Energy is thereby transferred to adjoining molecules, facilitating thermal conductivity. Thermal conductivity in Cu solid is achieved through the movement of free electrons between lattice structures. Copper is a better conductor of heat compared to other materials. As the Cu content increases, it exhibits significantly better heat dissipation in the 50-Cu sample. Correspondingly, we also used the model in Fig. 3D and F to simulate the heat transfer behavior of samples with Cu functional phases in different distribution states, as shown in Fig. 4C. It can be seen that the formation of Cu network not only provides a conductive network but also serves as a good thermal conductivity network, enabling Cu/CCTO materials to achieve controllable EN response while having excellent thermal conductivity [46]. This will provide important impetus for further promoting the application of metal/ceramic binary nanocomposites in new EM components and other fields [46–48].

## Conclusion

In conclusion, the as-prepared Cu/CCTO metacomposites have successfully achieved tunable ENZ and EN parameters within the 20-MHz to 1-GHz frequency range. The elucidation of the 2 dielectric response mechanisms responsible for negative permittivity, namely, electric dipole resonance and low-frequency plasmonic state, was further expounded upon through an analysis of the charge transport behavior at the Cu/CCTO interface constructed based on density functional theory. By integrating measured dielectric and thermal numerical values into multiple physical field models, the EM simulations demonstrated that the loss of EN materials can be converted into heat and dissipated through the formation of a 3D copper network. Our work proposes a universal design paradigm for typical binary metal/ceramic composite materials, providing novel ideas and research foundations for the application of EN and ENZ EM media in new sensors, shields, supercapacitors, and beyond.

## Materials and Methods

Details about the methods used in this work are available in the supplementary material.

## Data Availability

All data are available in the main text or the Supplementary Materials. Source data are available from the corresponding author upon reasonable request.

## References

[1] Xie P, Zhang Z, Wang Z, Sun K, Fan R. Targeted double negative properties in silver/silica random metamaterials by precise control of microstructures. Research. 2019;2019:1021368. 31549041 10.34133/2019/1021368 PMC6750100

[2] Reshef O, De Leon I, Alam MZ, Boyd RW. Nonlinear optical effects in epsilon-near-zero media. Nat Rev Mater. 2019;4(8):535–551.

[3] Sun K, Zhang Z, Tian J, Zeng N, Wang B, Xing W, Ma L, Long Y, Wang C, Fan R. Flexible and biocompatible polyurethane/Co@C composite films with weakly negative permittivity. Adv Compos Hybrid Mater. 2024;7(1):22.

[4] Lu X, Shapiro M, Mastovsky I, Temkin R, Conde M, Power J, Shao J, Wisniewski EE, Jing C. Generation of high-power, reversed-Cherenkov wakefield radiation in a metamaterial structure. Phys Rev Lett. 2019;122(1):014801–014804. 31012710 10.1103/PhysRevLett.122.014801

[5] Schurig D, Mock J, Justice B, Cummer S, Pendry J, Starr A, Smith D. Metamaterial electromagnetic cloak at microwave frequencies. Science. 2006;314(5801):977–980. 17053110 10.1126/science.1133628

[6] Leng Z, Yang Z, Tang X, Helal M, Qu Y, Xie P, El-Bahy Z, Meng S, Ibrahim M, Yu C, et al. Progress in percolative composites with negative permittivity for applications in electromagnetic interference shielding and capacitors. Adv Compos Hybrid Mater. 2023;6(1):195.

[7] Cheng C, Liu Y, Ma R, Fan R. Nickel/yttrium iron garnet metacomposites with adjustable negative permittivity behavior toward electromagnetic shielding application. Compos Part A Appl Sci Manuf. 2022;155: Article 106842.

[8] Shi Y, Fan G, Sun K, Hou Q, Wang Z, Liu Y, Fan R. Epsilon-negative media from the viewpoint of materials science. EPJ Appl Metamater. 2021;8:11.

[9] Xie P, Shi Z, Feng M, Sun K, Liu Y, Yan K, Liu C, Moussa TAA, Huang M, Meng S, et al. Recent advances in radio-frequency negative dielectric metamaterials by designing heterogeneous composites. Adv Compos Hybrid Mater. 2022;5(2):679–695.

[10] Sun L, Shi Z, He B, Wang H, Liu S, Huang M, Shi J, Dastan D, Wang H. Asymmetric trilayer all-polymer dielectric composites with simultaneous high efficiency and high energy density: A novel design targeting advanced energy storage capacitors. Adv Funct Mater. 2021;31(35):2100280.

[11] He Q, Sun K, Shi Z, Liu Y, Fan R. Polymer dielectrics for capacitive energy storage: From theories materials to industrial capacitors. Mater Today. 2023;68:298–333.

[12] Zhu L, Yang Y, Li Y, Shi Z, Bie X, Yuan Y, Fan R. Remarkably enhancing dielectric permittivity and suppressing loss of PVDF *via* incorporating metal nanoparticles decorated glass fibers. J Phy D Appl Phys. 2024;57(20): Article 205503.

[13] Cheng C, Jiang Y, Sun X, Shen J, Wang T, Fan G, Fan R. Tunable negative permittivity behavior and electromagnetic shielding performance of silver/silicon nitride metacomposites. Compos Part A Appl Sci Manuf. 2020;130: Article 105753.

[14] Fan G, Wang Z, Ren H, Liu Y, Fan R. Dielectric dispersion of copper/rutile cermets: Dielectric resonance, relaxation, and plasma oscillation. Scr Mater. 2021;190:1–6.

[15] Fan G, Wang Z, Zhang G, Liu Y, Fan R. Percolated cermets of nickel/yttrium iron garnet for double negative metacomposites. Compos Commun. 2021;24: Article 100667.

[16] Tian J, Fan R, Xie P, Wu H, Jiang S, Zhou Y, Sarychev AK. Flexible silver nanorods/carbon fiber felt metacomposites with epsilon-near-zero property adjusted by compressive deformation. Rare Metals. 2023;42(10):3318–3325.

[17] Qu Y, Xie P, Zhou Y, Ding J, Chen Y, Gong X, Yang J, Peng Q, Qi X. Graphitized-MWCNT/CaCu_3_Ti_4_O_12_ metacomposites for tunable ε′-negative and ε′-near-zero response with enhanced electromagnetic shielding. Ceram Int. 2023;49(23):37407–37414.

[18] Wang Z, Sun K, Xie P, Hou Q, Liu Y, Gu Q, Fan R. Design and analysis of negative permittivity behaviors in barium titanate/nickel metacomposites. Acta Mater. 2020;185:412–419.

[19] Wang Z, Sun K, Xie P, Liu Y, Gu Q, Fan R, Wang J. Epsilon-negative BaTiO_3_/Cu composites with high thermal conductivity and yet low electrical conductivity. J Mater. 2020;6(1):145–151.

[20] Sun K, Wang C, Tian J, Zhang Z, Zeng N, Yin R, Duan W, Hou Q, Zhao Y, Wu H, et al. Magnetic-driven broadband epsilon-near-zero materials at radio frequency. Adv Funct Mater. 2024;34(2):2306747.

[21] Wang Y, Wei Z, Song X, Liu M, Zeng Q, Jiang J, Liu Y, Fan R. Radio frequency epsilon-near-zero properties interpretation via CNT/PVDF composites. Appl Phys Lett. 2023;123(25): Article 251701.

[22] Qu Y, Zhou Y, Yang Q, Cao J, Liu Y, Qi X, Jiang S. Lignin-derived lightweight carbon aerogels for tunable epsilon-negative response. Adv Sci. 2024;11(11):2401767. 10.1002/advs.202401767 PMC11234391 38713745

[23] Qu Y, Zhou Y, Manshaii F, Wang K, Deng C, Liu Y. Three-dimensional random network of metacomposites by synergizing multi-walled carbon nanotube-carbon black for tunable epsilon-negative and epsilon-near-zero responses. Compos Part A Appl Sci Manuf. 2024;186: Article 108410.

[24] Song X, Shi G, Fan G, Liu Y, Fan R. Low-frequency plasmonic state and tunable negative permittivity in percolative graphite/barium titanate composites. Ceram Int. 2022;48(11):832–836.

[25] Wu H, Zhong Y, Tang Y, Huang Y, Liu G, Sun W, Xie P, Pan D, Liu C, Guo Z. Precise regulation of weakly negative permittivity in CaCu_3_Ti_4_O_12_ metacomposites by synergistic effects of carbon nanotubes and grapheme. Adv Compos Hybrid Mater. 2022;5:419–430.

[26] Smith DR, Padilla WJ, Vier DC, Nemat-Nasser SC, Schultz S. Composite medium with simultaneously negative permeability and permittivity. Phys Rev Lett. 2000;84:4184–4187. 10990641 10.1103/PhysRevLett.84.4184

[27] Ma R, Cheng C, Liu Y, Wang J, Zhou J, Hu Z, Gui H, Li J, Fan R. Temperature dependence of negative permittivity behavior in graphene/alumina ceramic metacomposites. J Eur Ceram Soc. 2024;44(5):3012–3019.

[28] Qu Y, Du Y, Fan G, Xin J, Liu Y, Xie P, You S, Zhang Z, Sun K, Fan R. Low-temperature sintering graphene/CaCu_3_Ti_4_O_12_ nanocomposites with tunable negative permittivity. J Alloy Compd. 2019;771:699–710.

[29] Qu Y, Wu J, Wang Z, Liu Y, Xie P, Wang Z, Tian J, Fan R. Radio-frequency epsilon-negative property and diamagnetic response of percolative ag/CCTO metacomposites. Scr Mater. 2021;203: Article 114067.

[30] Qu Y, Wang Z, Xie P, Wang Z, Fan R. Ultraweakly and fine-tunable negative permittivity of polyaniline/nickel metacomposites with high-frequency diamagnetic response. Compos Sci Technol. 2022;7:19092.

[31] Zhou Y, Qu Y, Yin L, Cheng W, Huang Y, Fan R. Coassembly of elastomeric microfibers and silver nanowires for fabricating ultra-stretchable microtextiles with weakly and tunable negative permittivity. Compos Sci Technol. 2022;223: Article 109415.

[32] Qu Y-P, Wu H-K, Xie P-T, Zeng N, Chen Y-L, Gong X, Yang J-L, Peng Q, Xie Y, Qi X-S. Carbon nanotube-carbon black/CaCu_3_Ti_4_O_12_ ternary metacomposites with tunable negative permittivity and thermal conductivity fabricated by spark plasma sintering. Rare Metals. 2023;42:4201–4211.

[33] Qu Y, Peng Q, Zhou Y, Manshaii F, Wang S, Wang K, Xie P, Qi X, Sun K. Fine-tunable ε′-negative response derived from low-frequency plasma oscillation in graphene/polyaniline metacomposites. Compos Commun. 2023;44: Article 101750.

[34] Fan G, Wang Z, Sun K, Liu Y, Fan R. Doped ceramics of indium oxides for negative permittivity materials in MHz-kHz frequency regions. J Mater Sci Technol. 2021;61:125–131.

[35] Xie P, Sun W, Du A, Hou Q, Wu G, Fan R. Epsilon-negative carbon aerogels with state transition from dielectric to degenerate semiconductor. Commun Electron Mater. 2021;7(3):2000877.

[36] Jiang Q, Xiang C, Luo Y, Wu L, Zhang Q, Zhao S, Qin F, Lin J-H. Textile structured metacomposites with tailorable negative permittivity under X and Ku band. Mater Des. 2020;185: Article 108270.

[37] Luo H, Qiu J. Carbon nanotube/polyolefin elastomer metacomposites with adjustable radio-frequency negative permittivity and negative permeability. Adv Electron Mater. 2019;5(6):1900011.

[38] Dai J, Jiang H, Guo Z, Qiu J. Tunable epsilon-and-mu-near-zero metacomposites. Adv Funct Mater. 2024;34(13):2308338.

[39] Zeng J, Xie W, Zhou H, Zhao T, Xu B, Jiang Q, Algadi H, Zhou Z, Gu H. Nitrogen-doped graphite-like carbon derived from phthalonitrile resin with controllable negative magnetoresistance and negative permittivity. Adv Compos Hybrid Mater. 2023;6:64.

[40] Shi S, Jiang T, Wang Y, Cao S, Gui X, Wu X, Li X, Li W, Yu J. Review on the construction of thermal conductive organic phase change materials by three-dimensional network method. J Mater Sci. 2023;58:13580–13604.

[41] Qu Y, Hao M, Luan X, Yang Q, Ding J, Zhou L, Liang G, Wang F, Xie P, Wu H. Regulation mechanism of epsilon-negative monolayer graphene/CaCu_3_Ti_4_O_12_ metacomposites for boosting electromagnetic shielding. Adv Compos Hybrid Mater. 2024;7:38.

[42] Qu Y-P, Zhou Y-L, Luo Y, Liu Y, Ding J-F, Chen Y-L, Gong X, Yang J-L, Peng Q, Qi X-S. Universal paradigm of ternary metacomposites with tunable epsilon-negative and epsilon-near-zero response for perfect electromagnetic shielding. Rare Metals. 2024;43:796–809.

[43] Liu X, Ren Z, Yang T, Hao Y, Wang Q, Zhou J. Tunable dielectric metamaterial based on strontium titanate artificial atoms. Scr Mater. 2020;184:30–33.

[44] Yang P, Sun K, Hou Q, Zheng H, Fan R. Sandwich-structured BaTiO_3_/ag ceramics embedded with a negative-epsilon layer to obtain high permittivity and suppress dielectric loss. J Eur Ceram Soc. 2024;44(4):2173–2181.

[45] Jing T, Xu K, Wang Y, Xiang L, Tang B, Shi S, Li Y, Yu W, Xie H, Wu X, et al. In situ construction of high-thermal-conductivity and negative permittivity epoxy/carbon fiber@carbon composites with a 3D network by high-temperature chemical vapor deposition. ACS Appl Mater Interfaces. 2023;15(46):54027–54038. 37938033 10.1021/acsami.3c15040

[46] Jiang T, Wang Y, Xu K, Xiang L, Tang B, Shi S, Wu X, Li W, Sun K, Fan R, et al. Highly thermally conductive and negative permittivity epoxy composites by constructing the carbon fiber/carbon networks. Compos Commun. 2023;39: Article 101560.

[47] Wei Z, Zhao L, Wang Z, Xu C, Zhang Y, Liu Y, Gao W, Fan R. Experimental observation of purely resistive effect in epsilon-near-zero transition metal perovskite. Acta Mater. 2024;266: Article 119704.

[48] Wei Z, Liu Y, Zhang Y, Aleksanteri KL, Qi X, Zhang Z, Cao W, Fan R. Negative correlation between thermal and electrical conductivity in epsilon-negative nanocomposites. Adv Electron Mater. 2023;10(4):2300614.

