# Supplementary material for: Microscopic Response Mechanism of Epsilon-Negative and Epsilon-Near-Zero Metacomposites
Source: Research (Wash D C). 2025 Feb 1;8:0556. doi: 10.34133/research.0556 (PMC11694408; doi:10.34133/research.0556)
Supplement: Supplementary 1 — Supplementary Text Figs. S1 to S41 Movie S1 [file research.0556.f1.zip › Supporting Information-REVISED.docx]

**Supporting Information**

Microscopic response mechanism of Epsilon-Negative and Epsilon-Near-Zero Metacomposites

Yunlei Zhou^a, b,^ *, Yanan Wang^a^, Shicheng Qiu^c^, Wei Zhao^a^, Shaolei Wang^c^, Hong Bao^a, b^, Yunpeng Qu^d,^ *, Zhen Wen ^e,^ *

^a^ Hangzhou Institute of Technology, Xidian University, Hangzhou, 311231, China

^b^ School of Mechano-Electronic Engineering, Xidian University, Xi’an710071, China

^c^ Department of Bioengineering, University of California, Los Angeles, Los Angeles, California, 90095, United States

^d^ College of Physics, Guizhou University, Guiyang 550025, China

^e^ Institute of Functional Nano and Soft Materials (FUNSOM), Jiangsu Key Laboratory for Carbon-Based Functional Materials & Devices, Soochow University, Suzhou 215123, P.R. China.

*Corresponding author: [zhouyunlei@xidian.edu.cn](mailto:zhouyunlei@xidian.edu.cn) (Y. Zhou); [ypqu@gzu.edu.cn](mailto:ypqu@gzu.edu.cn)(Y. Qu); [wenzhen2011@suda.edu.cn](mailto:wenzhen2011@suda.edu.cn) (Z. Wen)

1. **Experimentation**

**1.1. Fabrication of Cu/CCTO Metacomposites**

Materials such as copper (Cu, >99.9%), CaCO_3_ (>99.9%), CuO (>99.9%), TiO_2_ (>99.9%), and polyvinyl alcohol (PVA, >99.99%) were purchased from Sinopharm Chemical Reagent Co., Ltd. The CCTO powders are prepared according to:

CaCO_3_ + 3CuO+ 4TiO_2_ → CaCu_3_Ti_4_O_12_ + CO_2_↑ (1)

where the chemical reaction occurs under the solid-state after stoichiometrically mixing by ball milling for 2 hours. The calcined process is conducted on 900 ^o^C in air condition and sustains 6 hours, during which the grain size of CCTO is fully grown. Then, according to the traditional sintering process of composite ceramics, we thoroughly mix Cu and CCTO in a mass ratio of 0-60 wt% and press them into the embryo. During this process, we use PVA with a concentration of 5% as a binder, and then sinter the embryo at 1000 ^o^C for 2 hours, using Ar gas as a protective atmosphere. After successful sintering, the sample undergoes surface polishing and grinding, and is subjected to dielectric property testing and structural and morphological characterization in the form of a diameter of 15mm and a thickness of 2mm.

**1.2. Dielectric Tests and Characterization**

The characterization techniques including field-emission scanning electron microscopy (FE-SEM, SU-70) with energy dispersive spectrometer (EDS), X-ray diffractometer (XRD, X’Pert Pro), transmission electron microscopy (TEM, JEOL-1230), and thermal gravimetric and differential scanning calorimetry (TG-DSC, STA499F3, Netzsch) were employed to investigate the microstructural evolution and compositions of Cu/CCTO composites. The Computerized Tomography (CT, YXLON FF35, Germany) technology characterizes the dual continuous distribution of the internal metal network structure and ceramic matrix in metacomposites. The dielectric properties such as the complex permittivity (*ε'*, *ε''*), AC conductivity (σ_ac_), loss tangent angle (tan*δ* = |*ε''*/*ε'*|), complex impedance (*Z'*, *Z''*), impedance modulus (|*Z*|) and phase angle (*φ*) were measured on a Precise Impedance Analyzer (Agilent E4991A, USA) equipped with a 16453A fixture. For the permittivity measurements, the samples were processed into a square disc with dimensions of 20 mm $\times$ 2 mm. After the open/short compensation (to reduce the residual impedances of the test fixtures) and load compensation (to calibrate the test fixture) the disc was put between the two planar electrodes of the 16453A dielectric test fixture for permittivity measurements. Besides, nonlinear fitting for dielectric data was performed in OriginPro 2024 software, while equivalent circuit analysis for impedance data was performed in ZsimWin software. An infrared thermal imager (H21pro, Hikvision) was used to evaluate the heat dissipation performance of Cu/CCTO composites at different environmental temperatures.

**1.3. First-Principles Calculations and Simulations**

The density functional theory (DFT) calculations were performed in Vienna ab initio simulation package (VASP). The CaTi_4_(CuO_4_)_3_@Cu calculations were executed within slab model, including CaTi_4_(CuO_4_)_3_ (111) layer and Cu layer, and the lattice constant mismatch is less than the 5% of what is typically required for calculating heterojunctions. The Fermi level (E_F_), work function, differential charge density and density of states (DOS) of this model were provided after structural optimization.

COMSOL Multiphysics was utilized to compute the potential distribution and thermal field distribution of Cu particles in two states: isolated distribution and network structure. The electrical parameters and thermal conductivity of Cu, CCTO and Ag electrodes were obtained from ideal material properties within the COMSOL software framework. When establishing the COMSOL multi physics field model, for the sake of simplifying the model and computational complexity, we used a regular arrangement to construct the distribution model of Cu particles, but their isolated distribution and network structure are consistent, which can explain our problem. The EM shielding effectiveness of Cu/CCTO metacomposites with EN and ENZ responses were presented by electric field vector distribution which also performed in COMSOL software. All dielectric parameters were taken from emprical measurements, with a standardized sample thickness of 1mm and periodic boundary conditions tailored to meet the software's modeling prerequisites. The input and output ends of the EM wave are set, and the sample is placed in the center of the model. These comprehensive analyses not only validate the percolation effect caused by structural evolution of the Cu/CCTO metacomposites but also facilitate demonstrating the ENZ and EN response mechanisms.


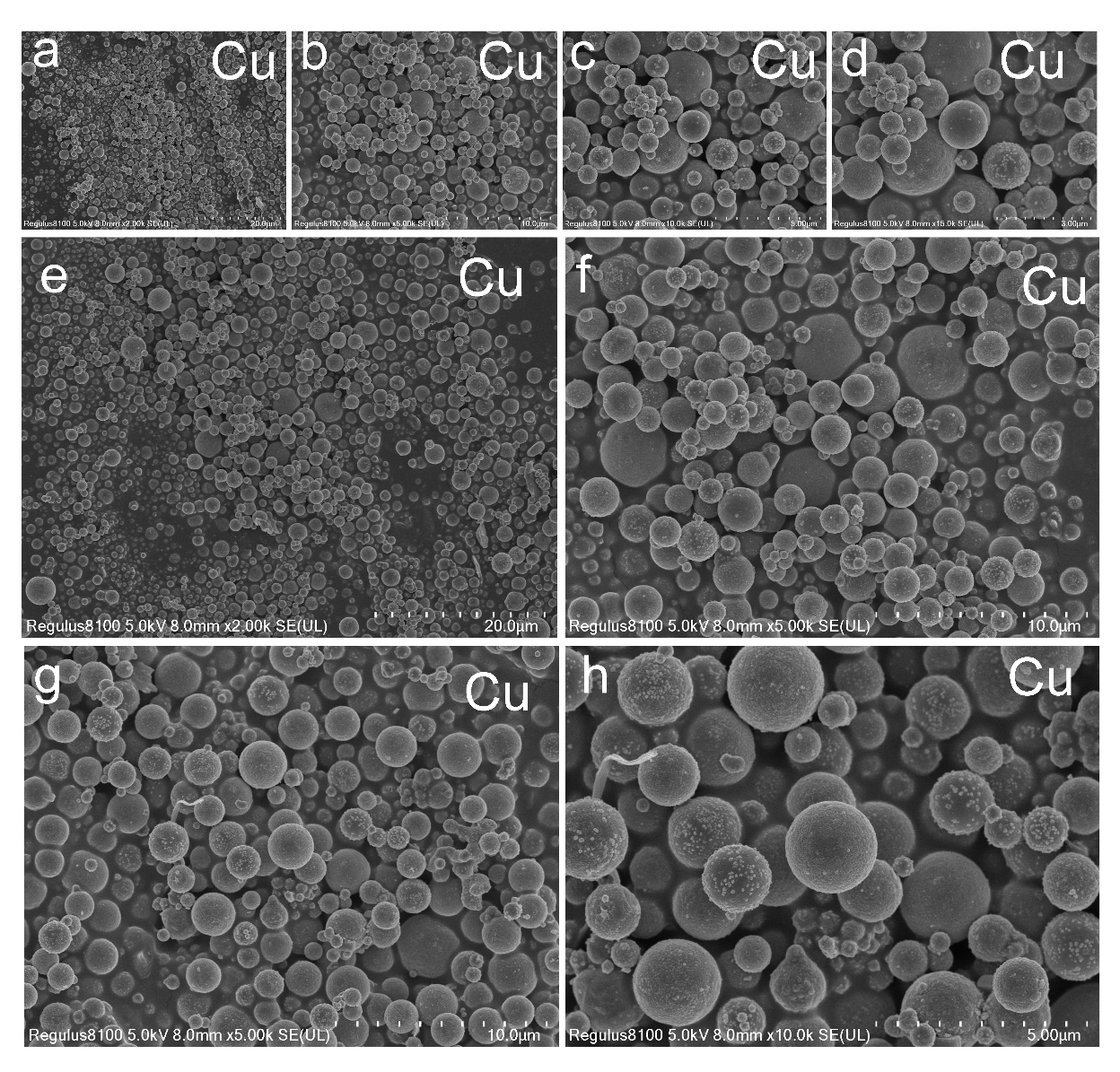
**Fig. S1.** FESEM images of Cu powders (a-h).


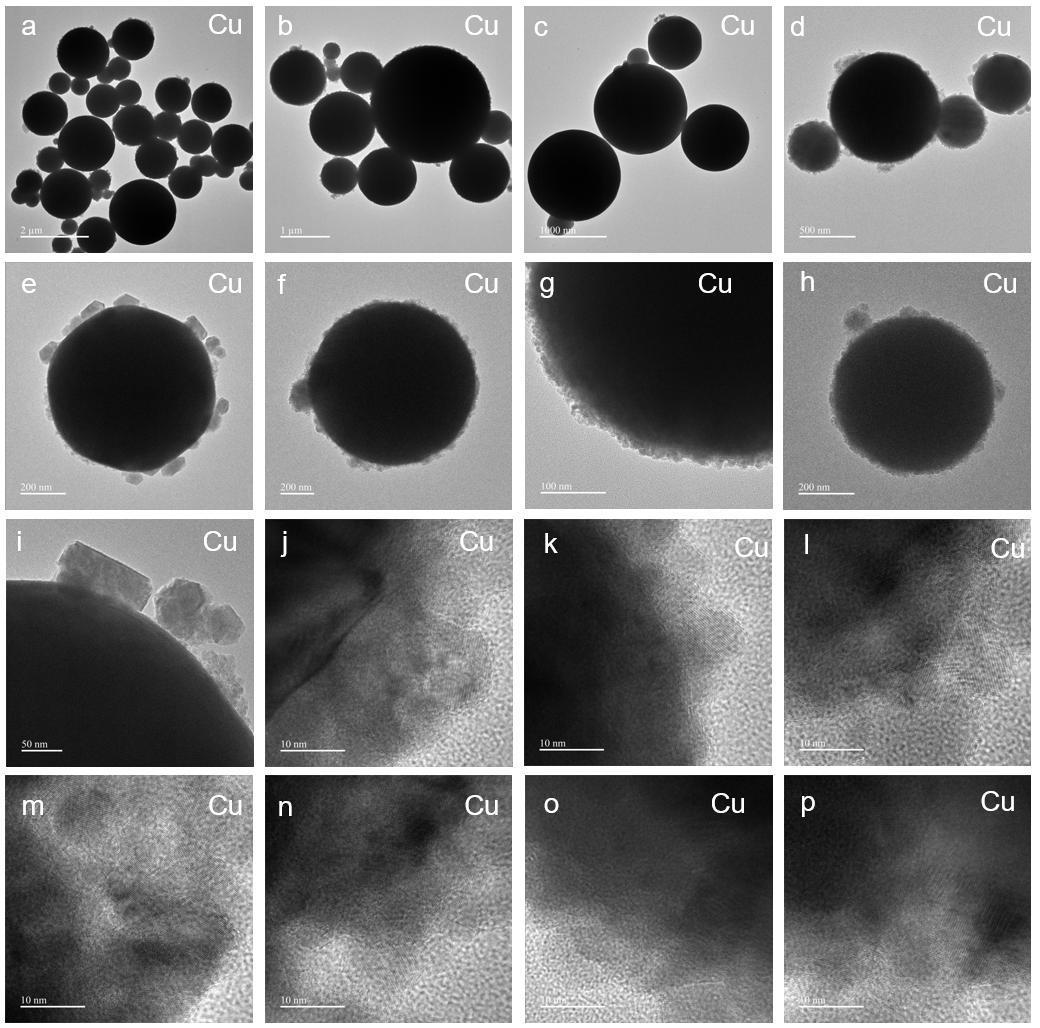


**Fig. S2.** TEM images of Cu powders (a-p).


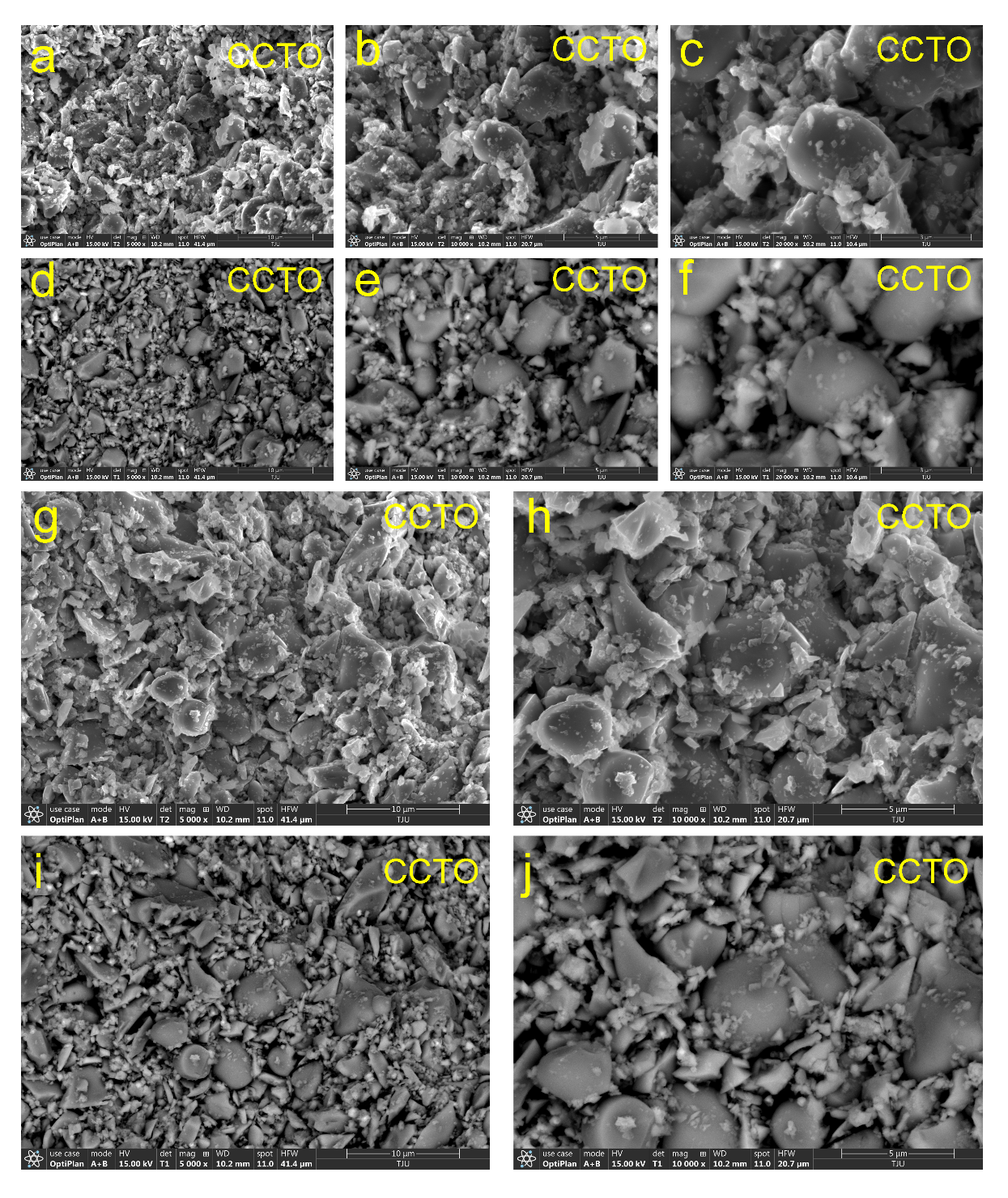
**Fig. S3.** FESEM images of CCTO after sintering (a-j).


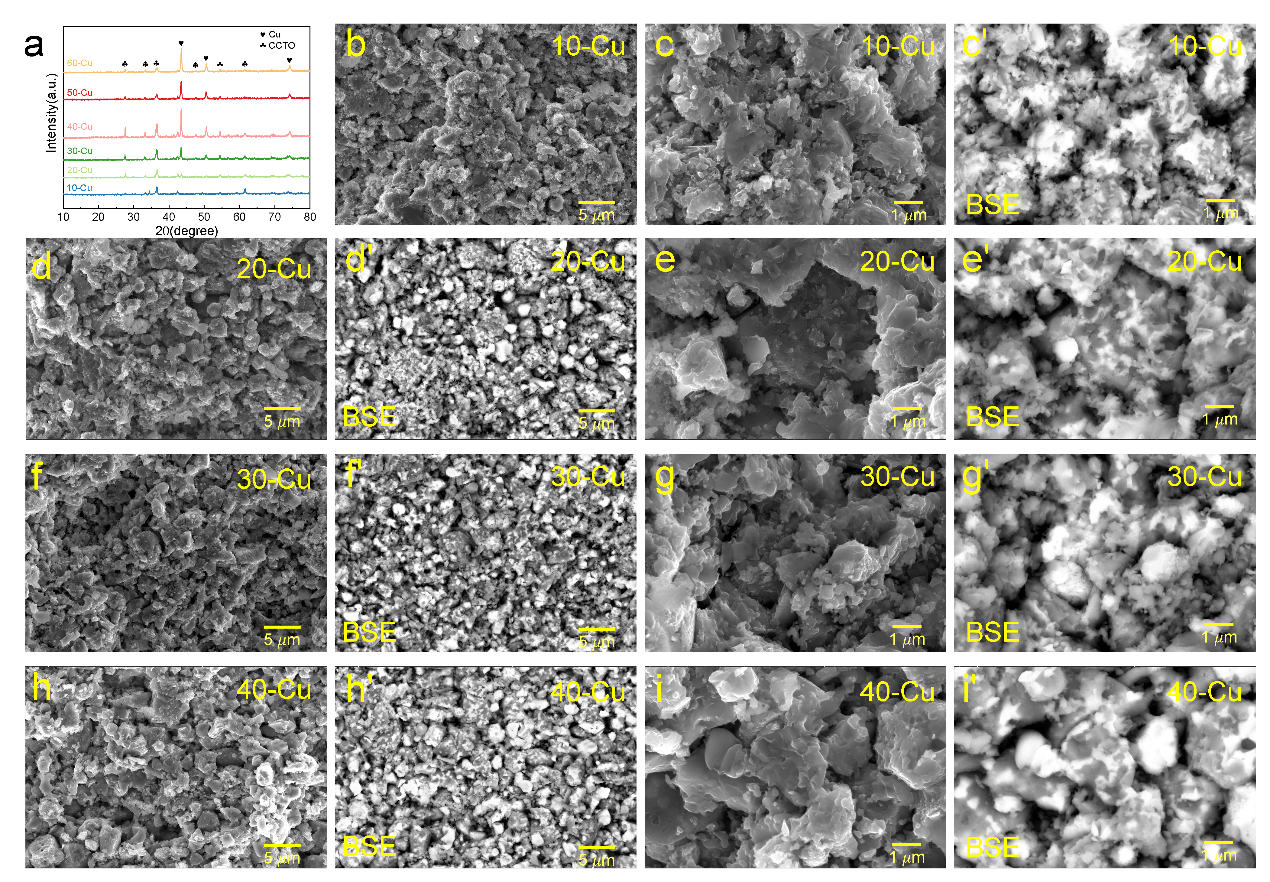
**Fig. S4.** XRD patterns of Cu/CCTO composites (a). FESEM and BSE images of Cu/CCTO composites with different Cu content (b-i').

**
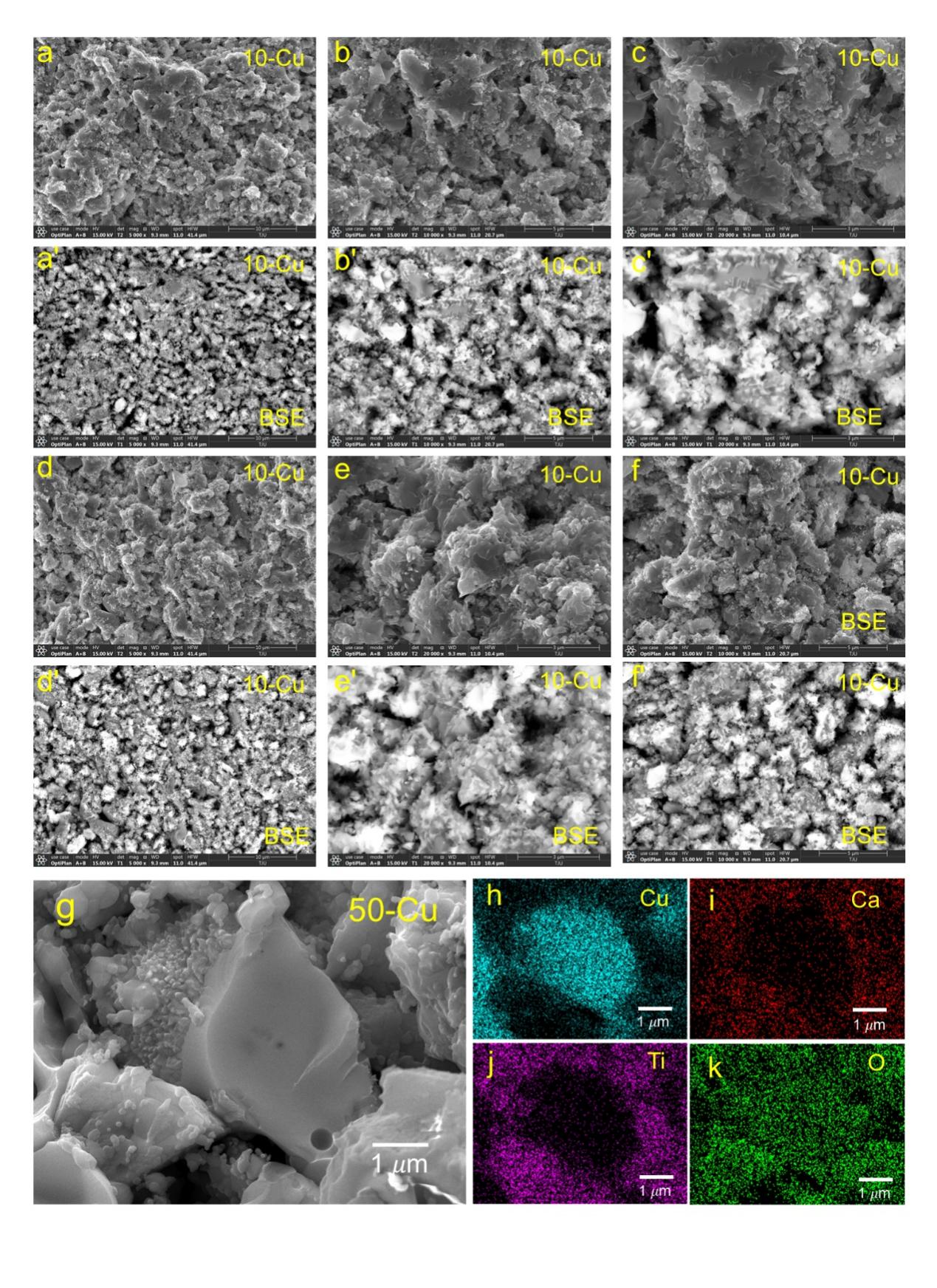
**

**Fig. S5.** FESEM, BSE and EDS images of Cu/CCTO composites with Cu content of 10 wt% (a-k).


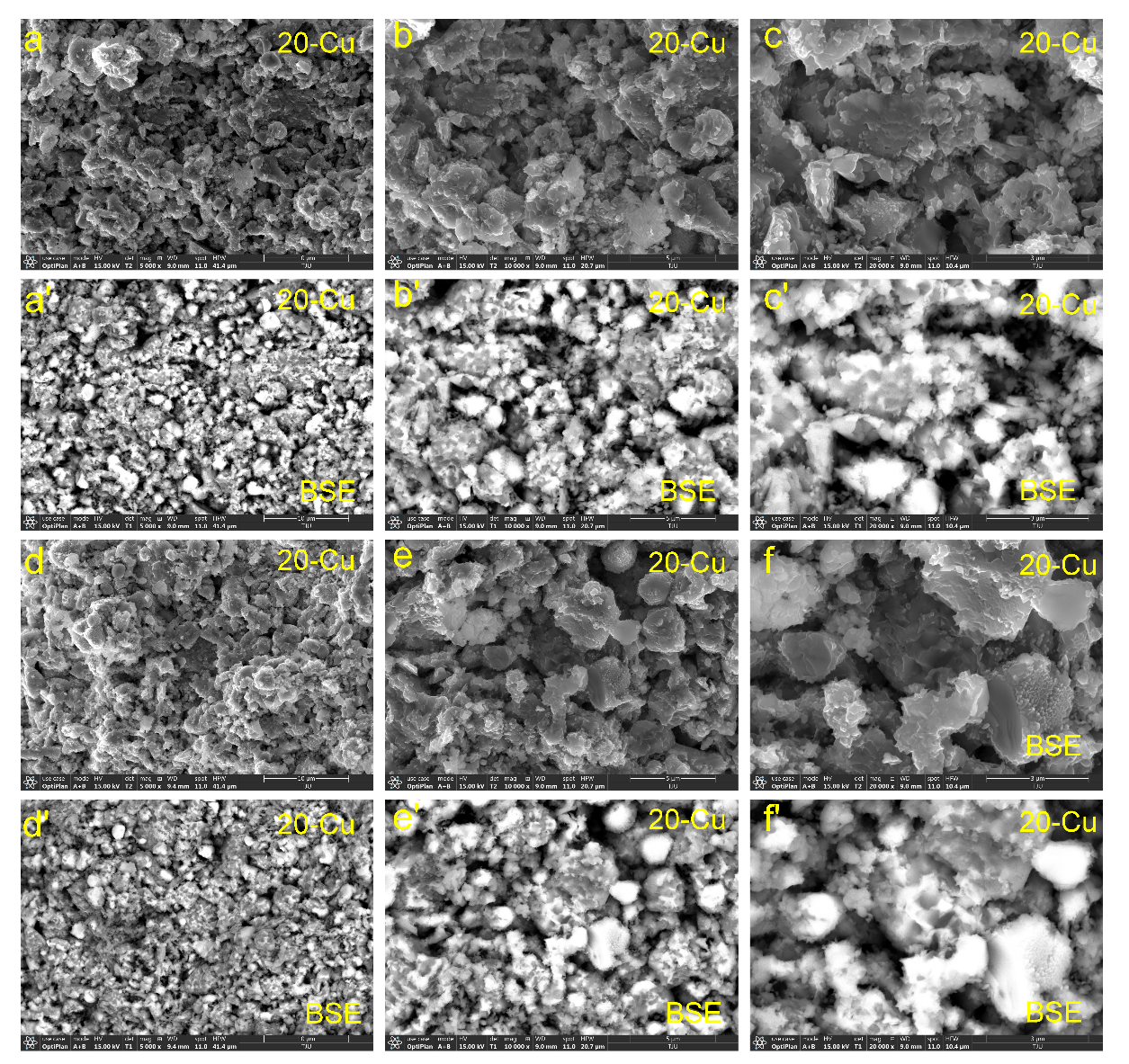
**Fig. S6.** FESEM and BSE images of Cu/CCTO composites with Cu content of 20 wt% (a-f').


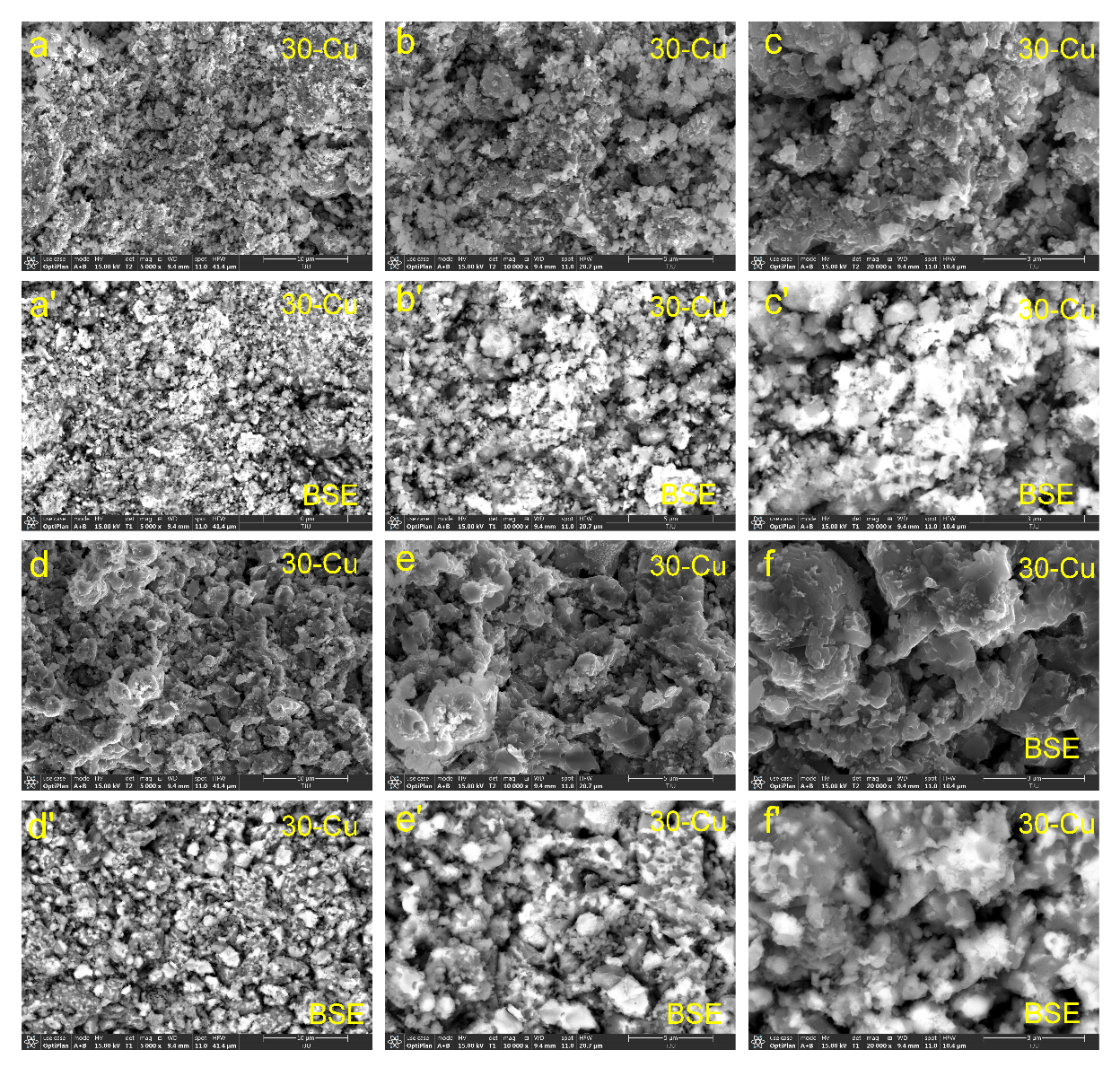
**Fig. S7.** FESEM and BSE images of Cu/CCTO composites with Cu content of 30 wt% (a-f').


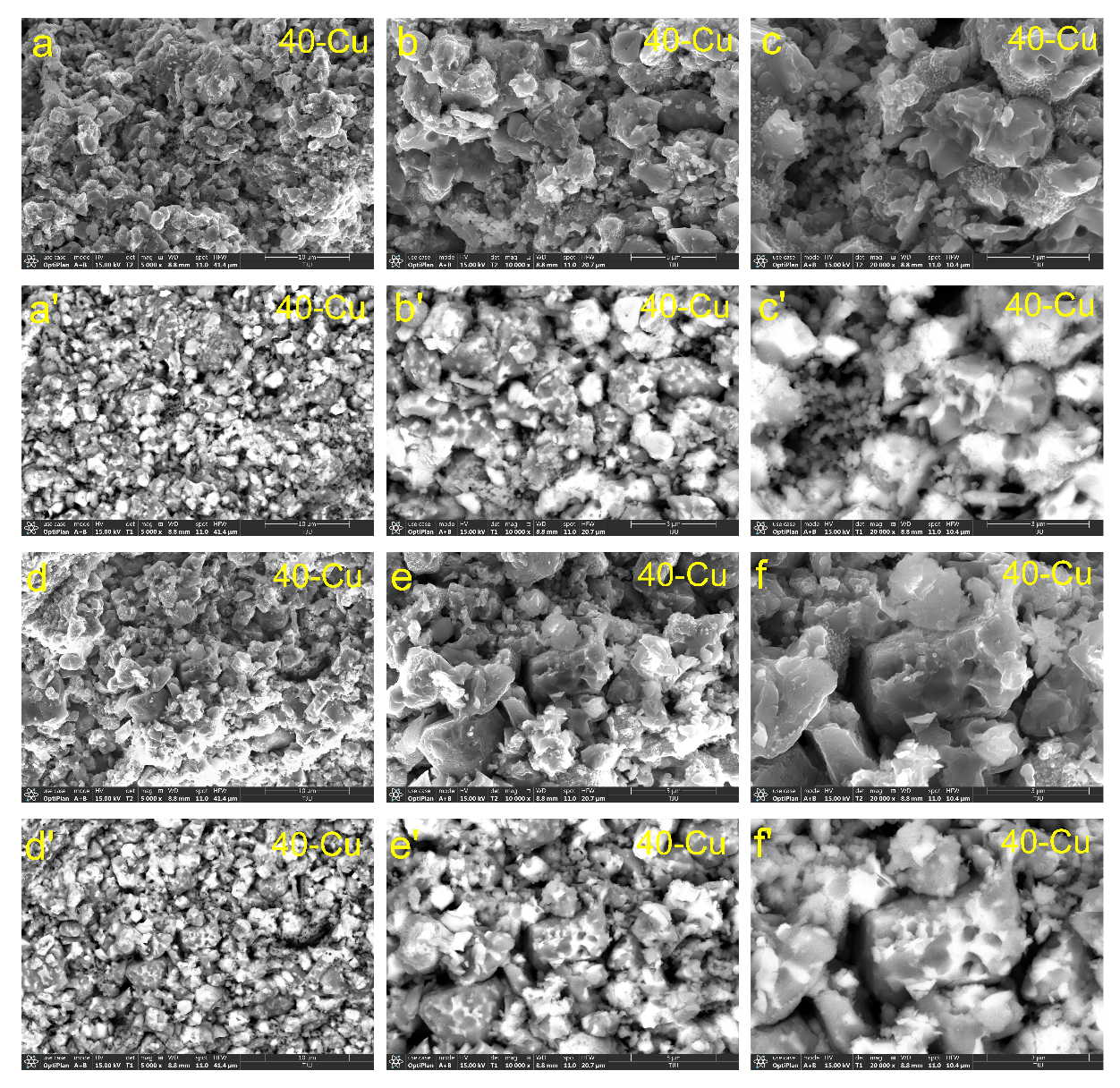
**Fig. S8.** FESEM and BSE images of Cu/CCTO composites with Cu content of 40 wt% (a-f').


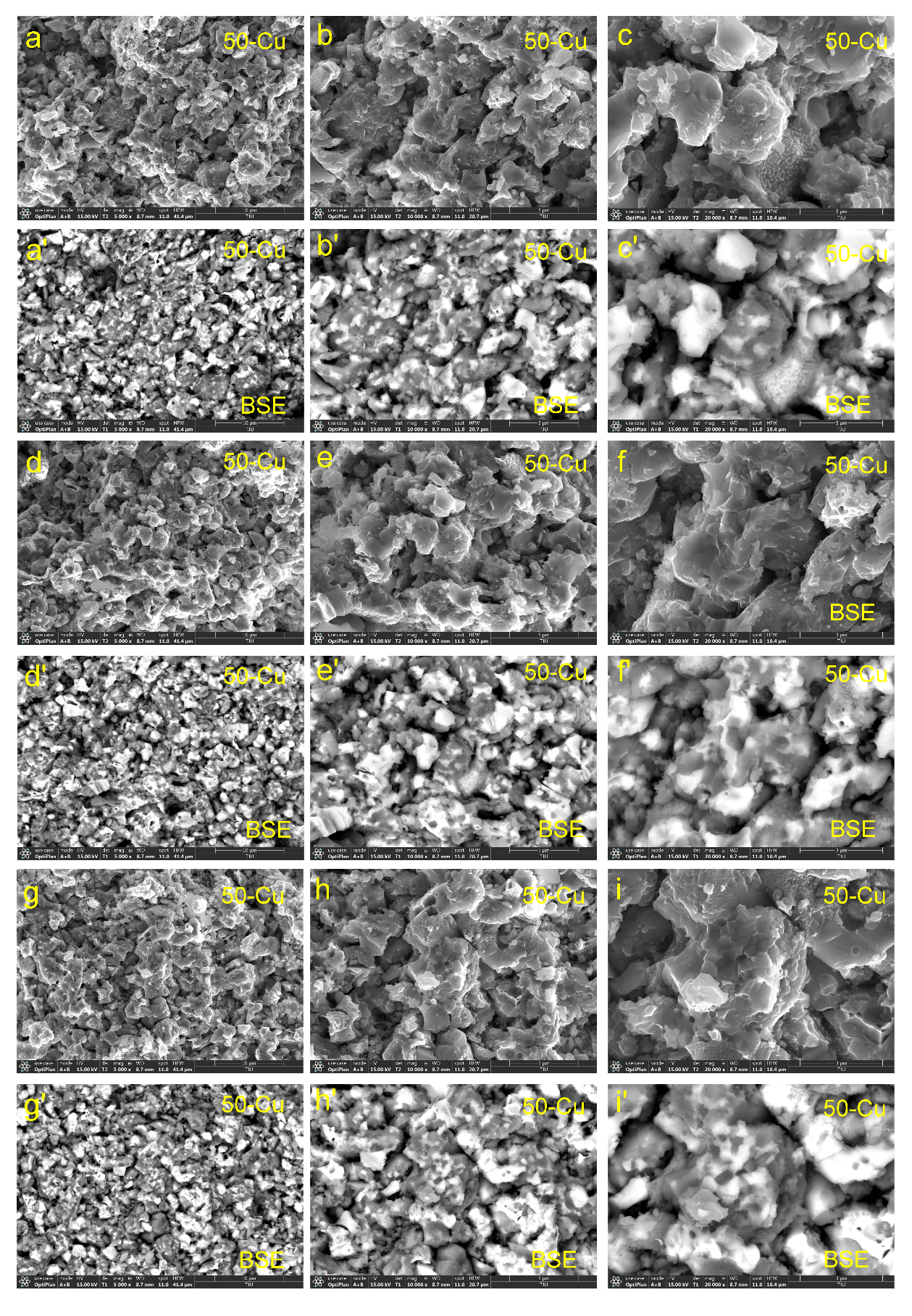
**Fig. S9.** FESEM and BSE images of Cu/CCTO composites with Cu content of 50 wt% (a-f').


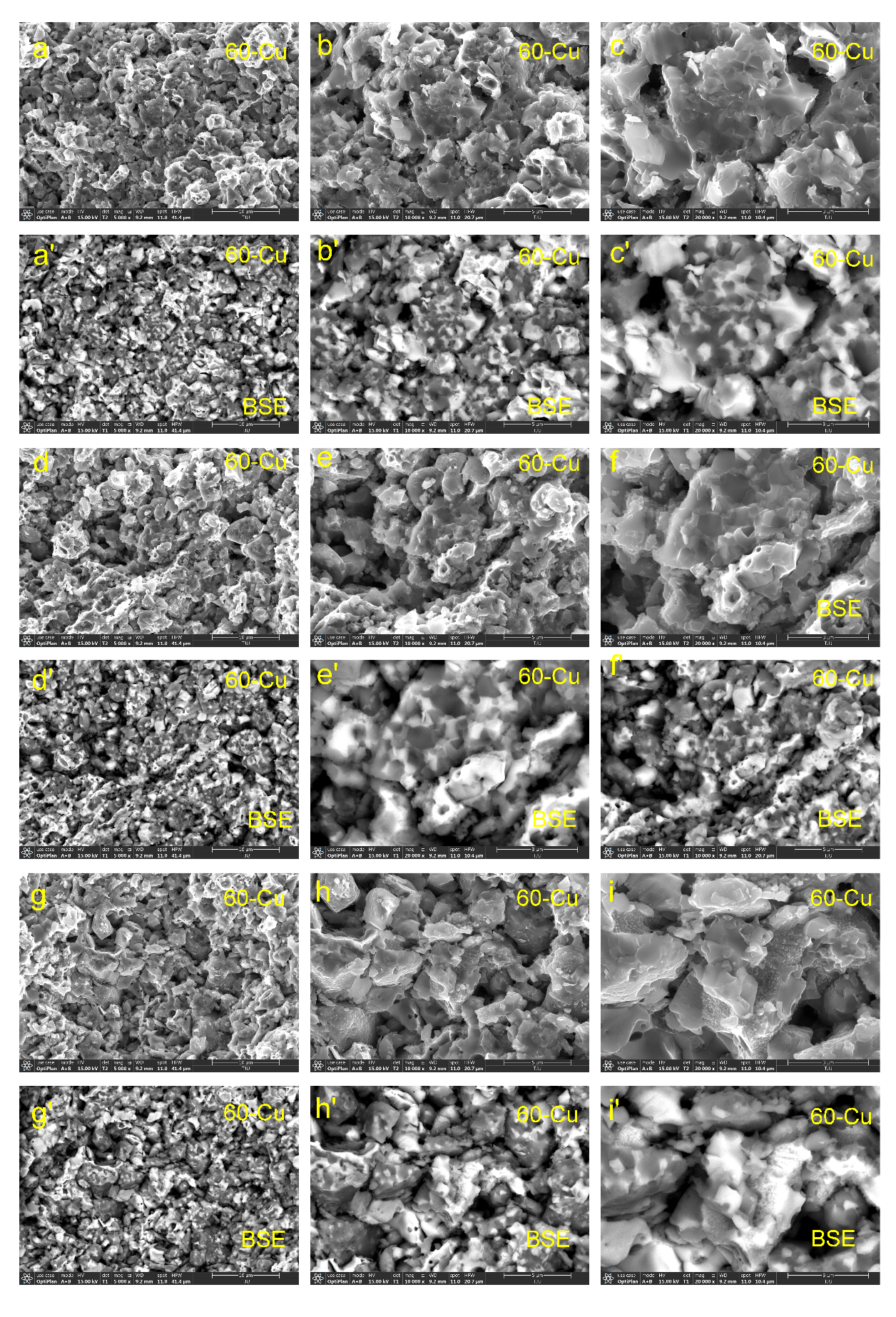
**Fig. S10.** FESEM and BSE images of Cu/CCTO composites with Cu content of 60 wt% (a-f').


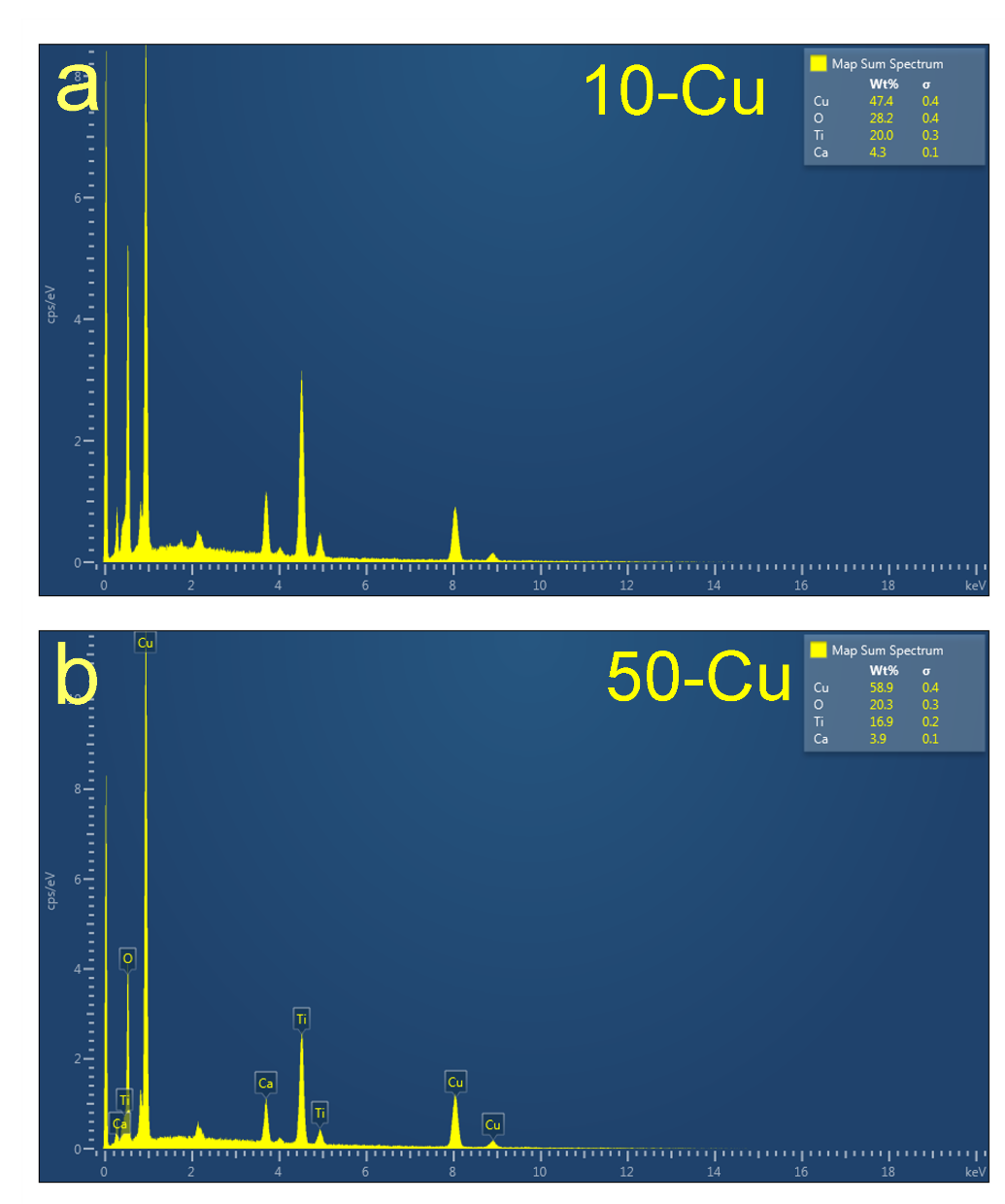


**Fig. S11.** Element content statistical chart of eds energy spectrum for composites with Cu content of 10 wt% and 50 wt% (a, b).


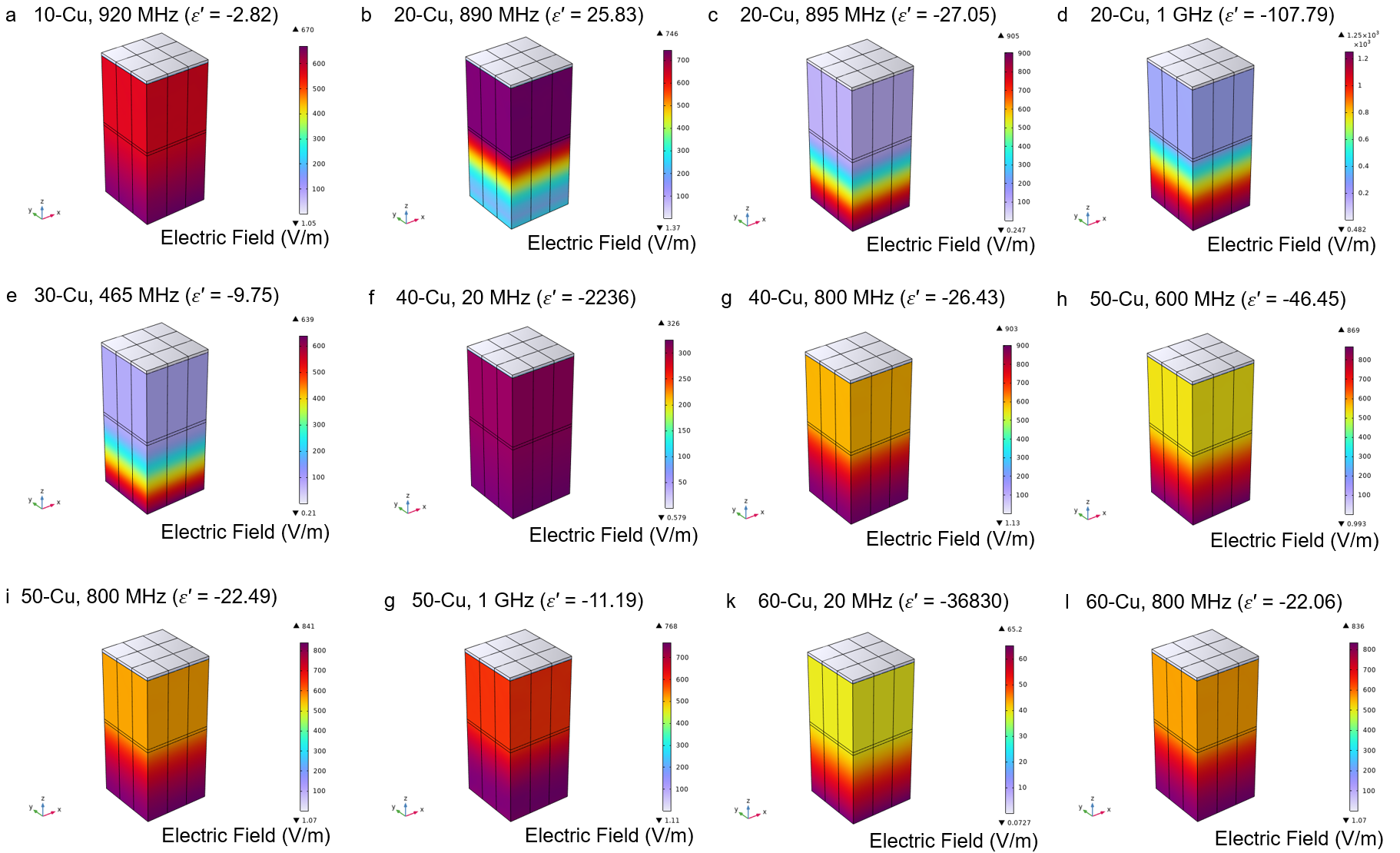
**Fig. S12.** Electric field vector distribution at different EN and ENZ frequencies, highlighting variations in Cu content (a-l).


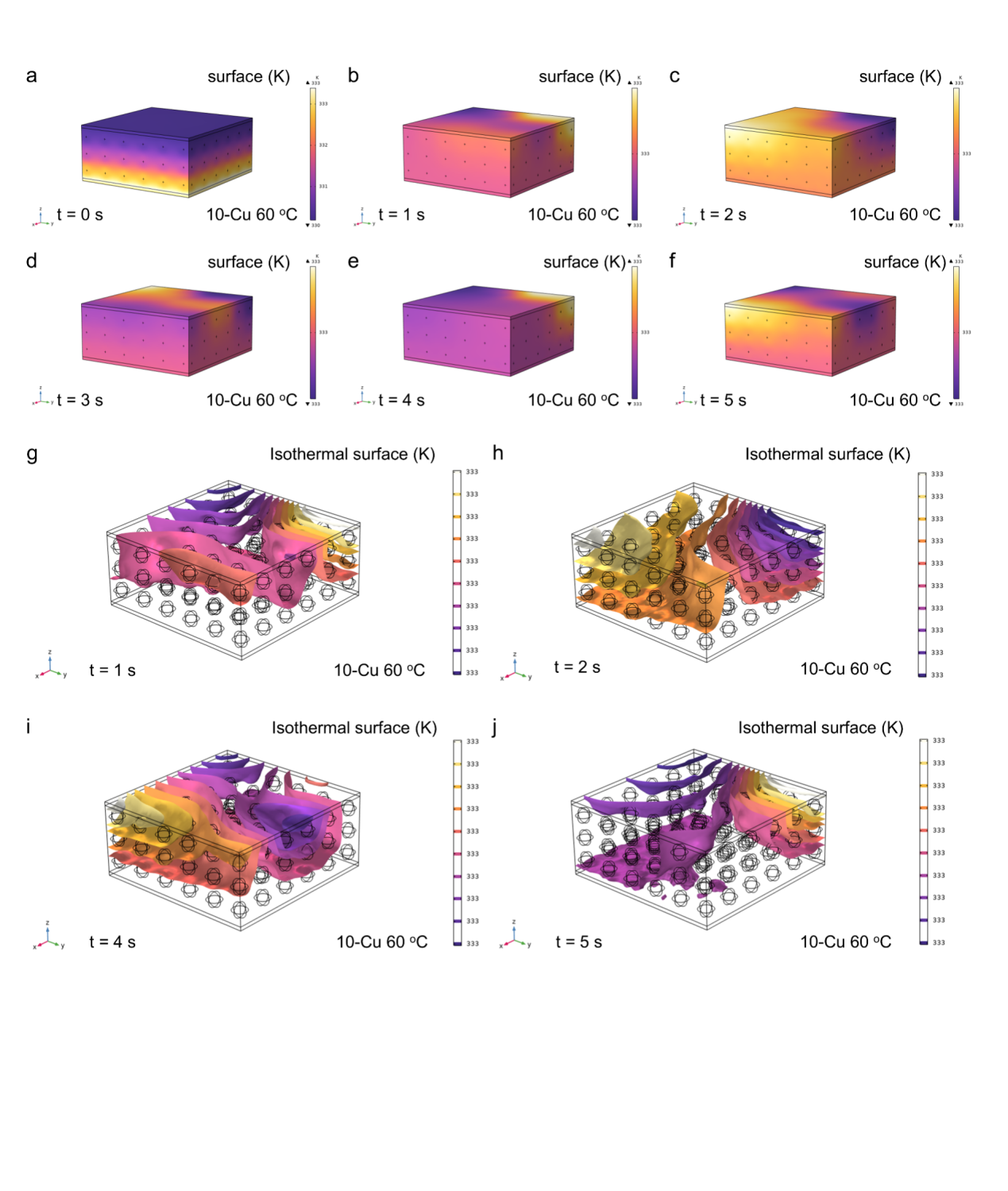


**Fig. S13.** Simulation diagram of thermal field distribution performed in COMSOL Software for 10-Cu sample (a-j).


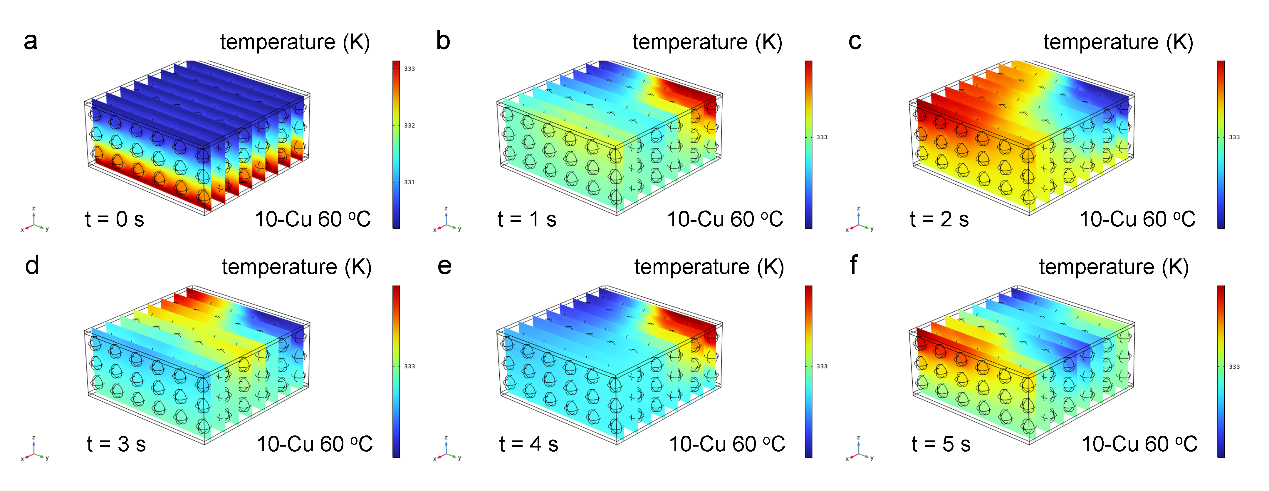
**Fig. S14.** Simulation diagram of thermal field distribution performed in COMSOL Software for 10-Cu sample (a-f).

**
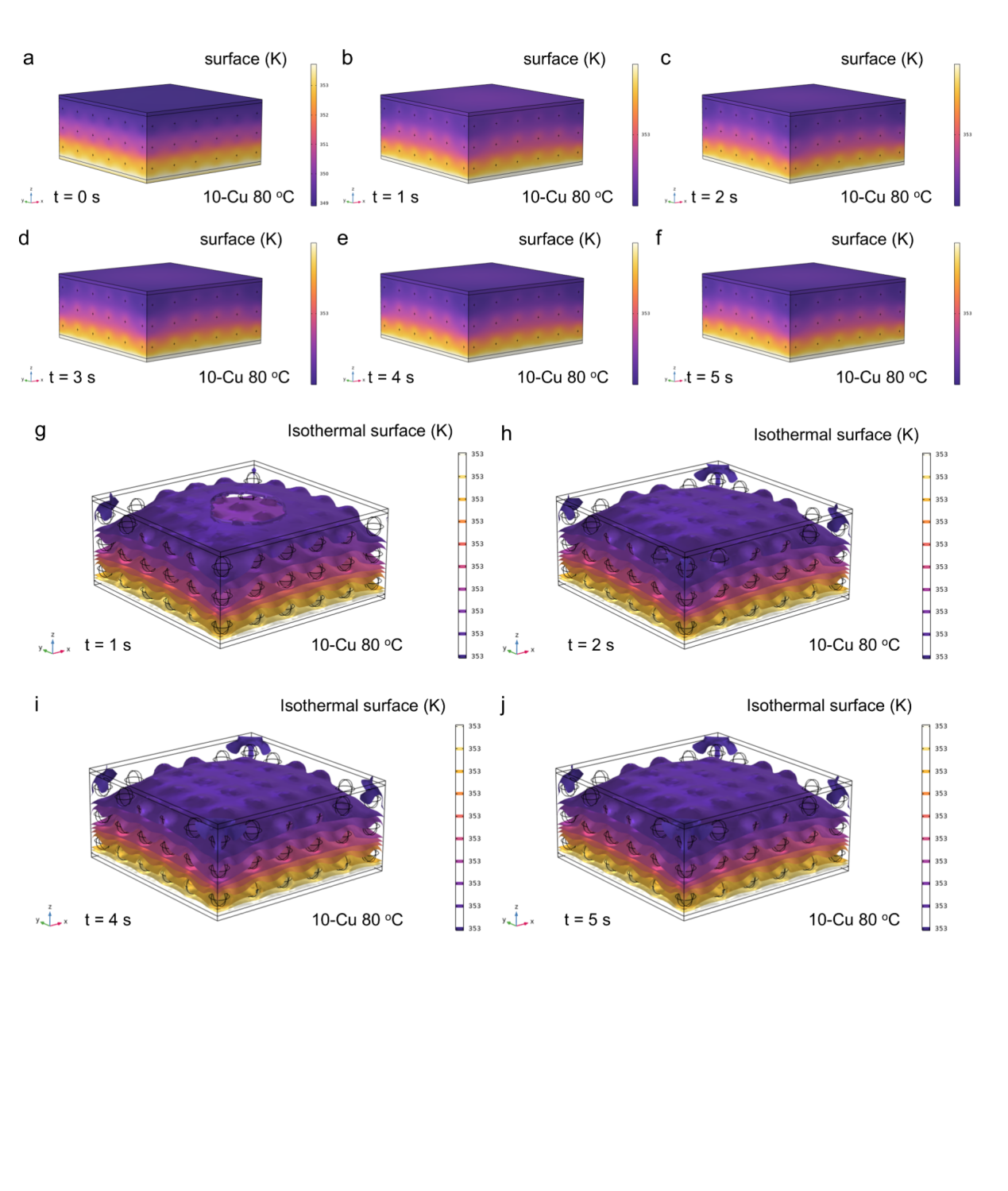
**

**Fig. S15.** Simulation diagram of thermal field distribution performed in COMSOL Software for 10-Cu sample (a-j).


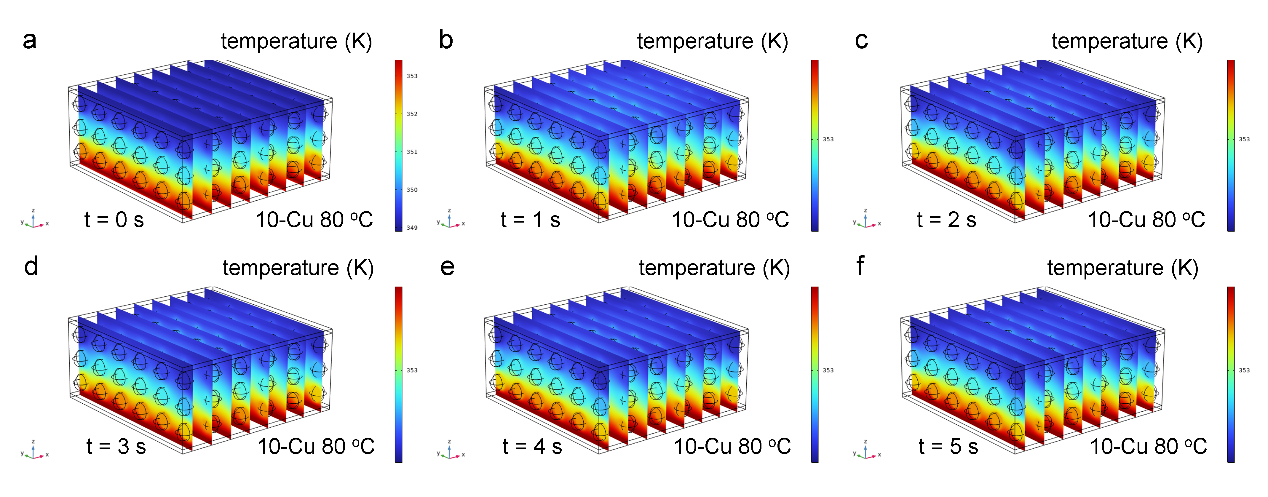
**Fig. S16.** Simulation diagram of thermal field distribution performed in COMSOL Software for 10-Cu sample (a-f).


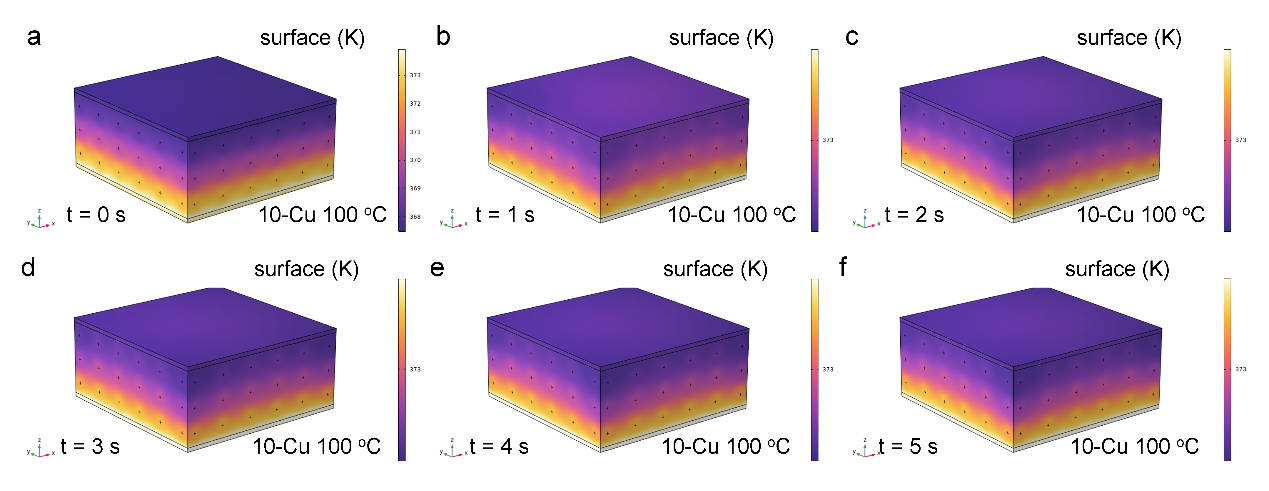
**Fig. S17.** Simulation diagram of thermal field distribution performed in COMSOL Software for 10-Cu sample (a-f).


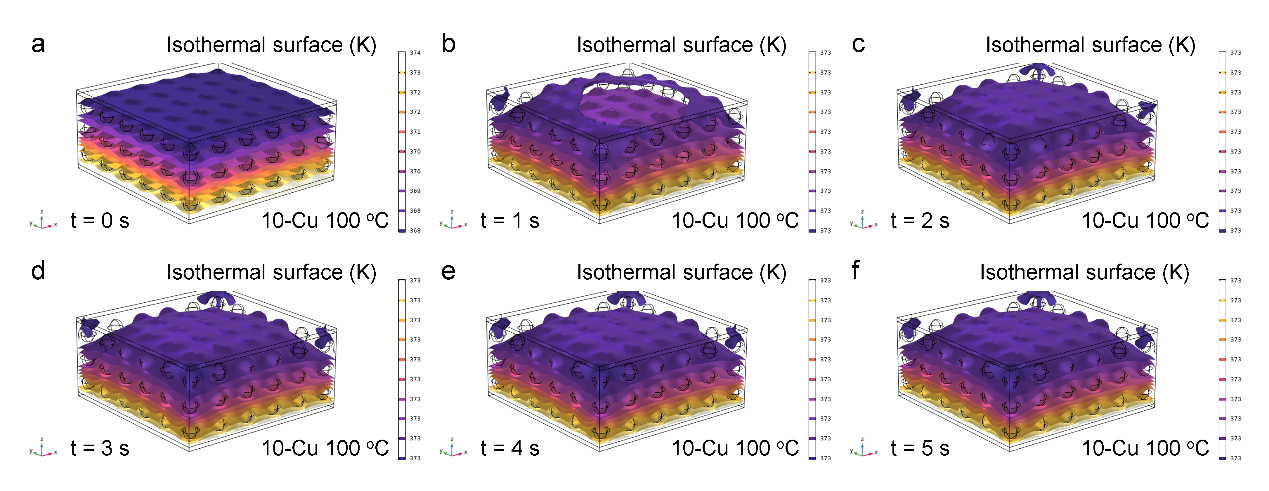
**Fig. S18.** Simulation diagram of thermal field distribution performed in COMSOL Software for 10-Cu sample (a-f).


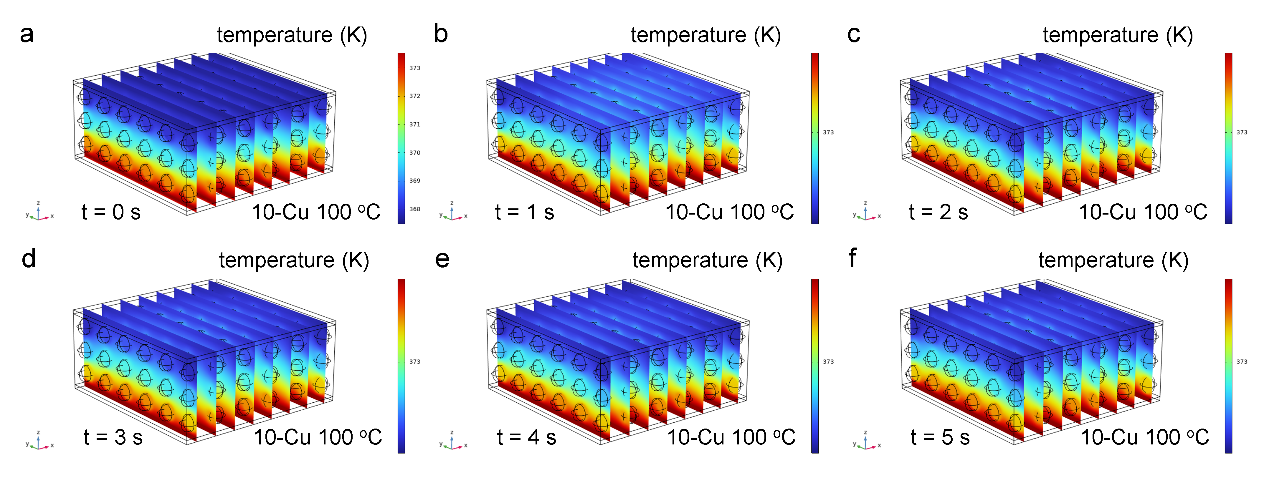
**Fig. S19.** Simulation diagram of thermal field distribution performed in COMSOL Software for 10-Cu sample (a-f).


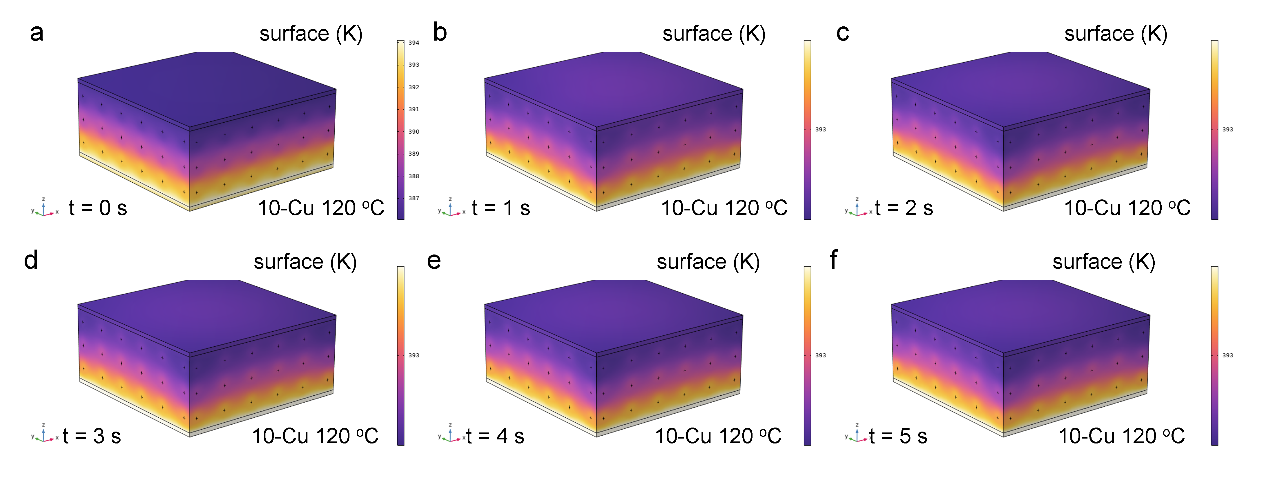
**Fig. S20.** Simulation diagram of thermal field distribution performed in COMSOL Software for 10-Cu sample (a-f).


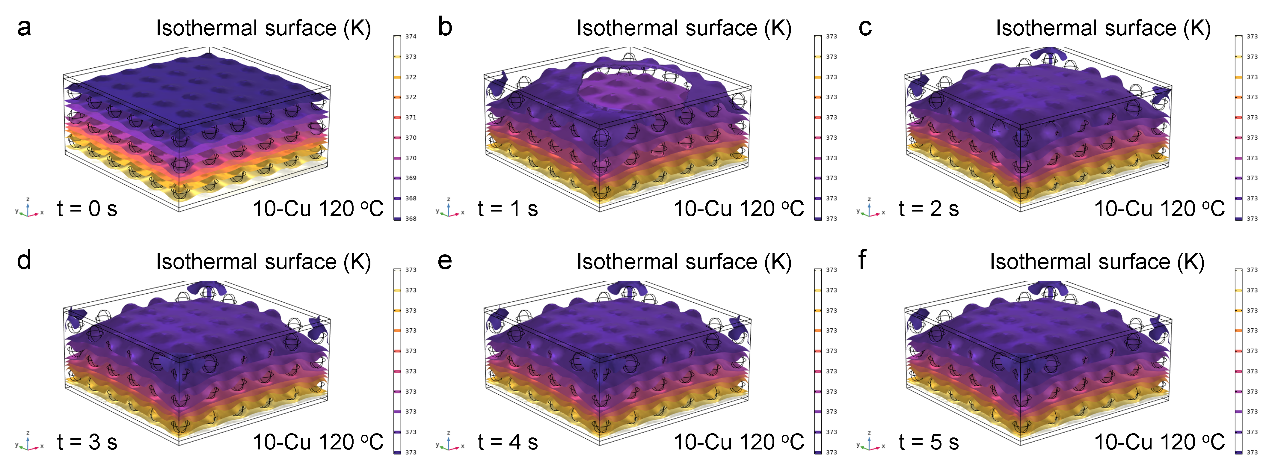
**Fig. S21.** Simulation diagram of thermal field distribution performed in COMSOL Software for 10-Cu sample (a-f).


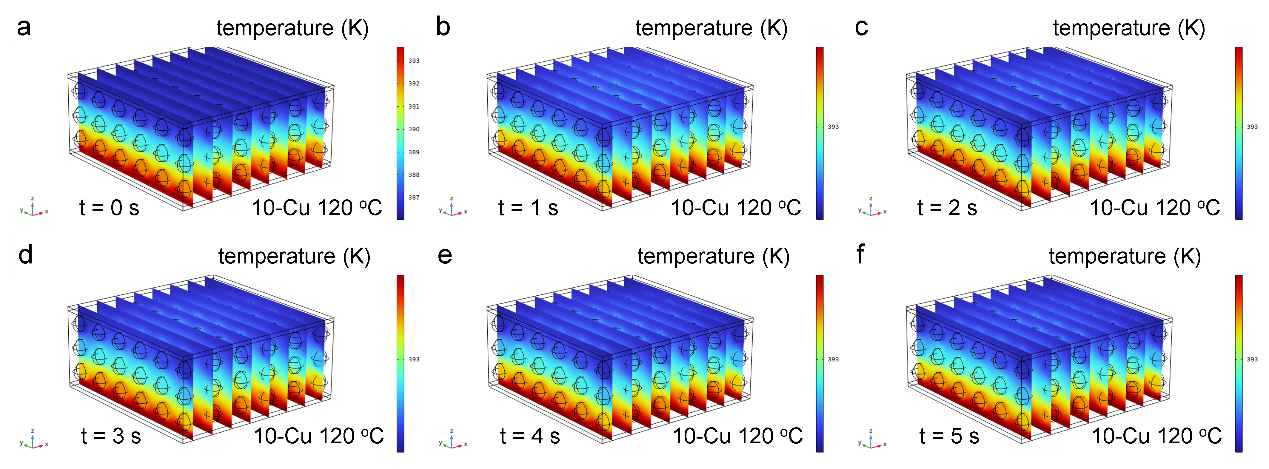
**Fig. S22.** Simulation diagram of thermal field distribution performed in COMSOL Software for 10-Cu sample (a-f).

**
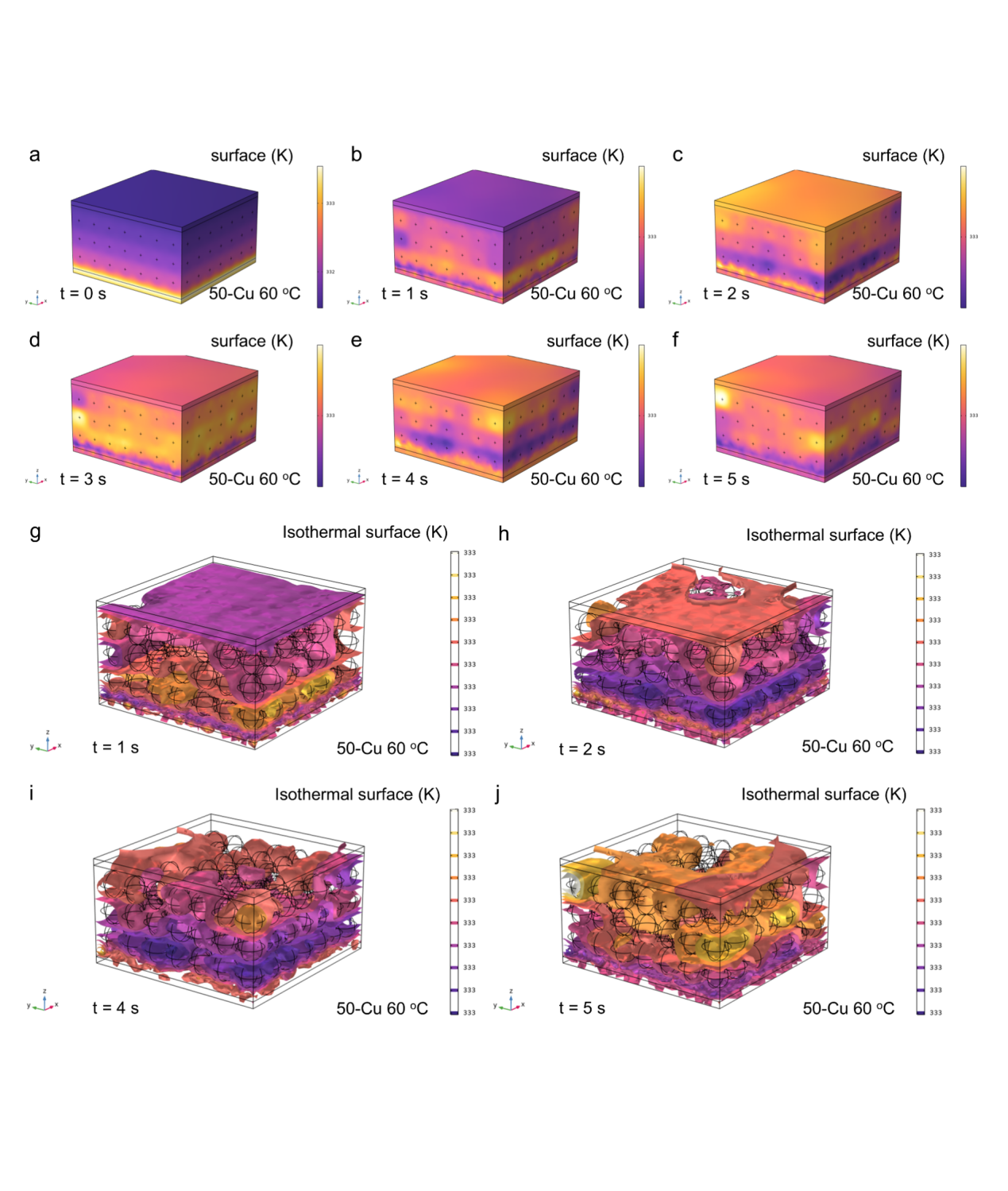
**

**Fig. S23.** Simulation diagram of thermal field distribution performed in COMSOL Software for 50-Cu sample (a-j).


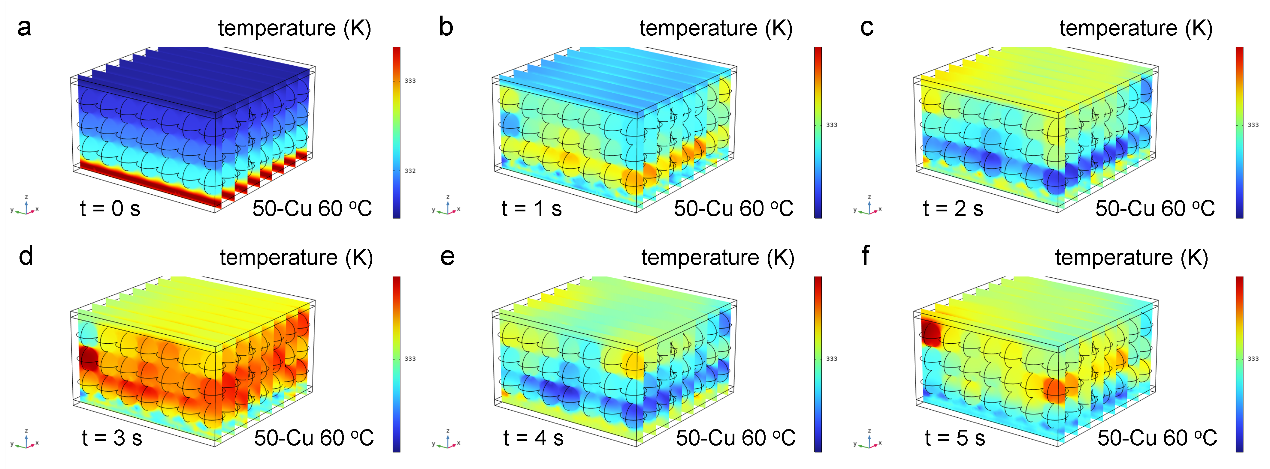
**Fig. S24.** Simulation diagram of thermal field distribution performed in COMSOL Software for 50-Cu sample (a-f).


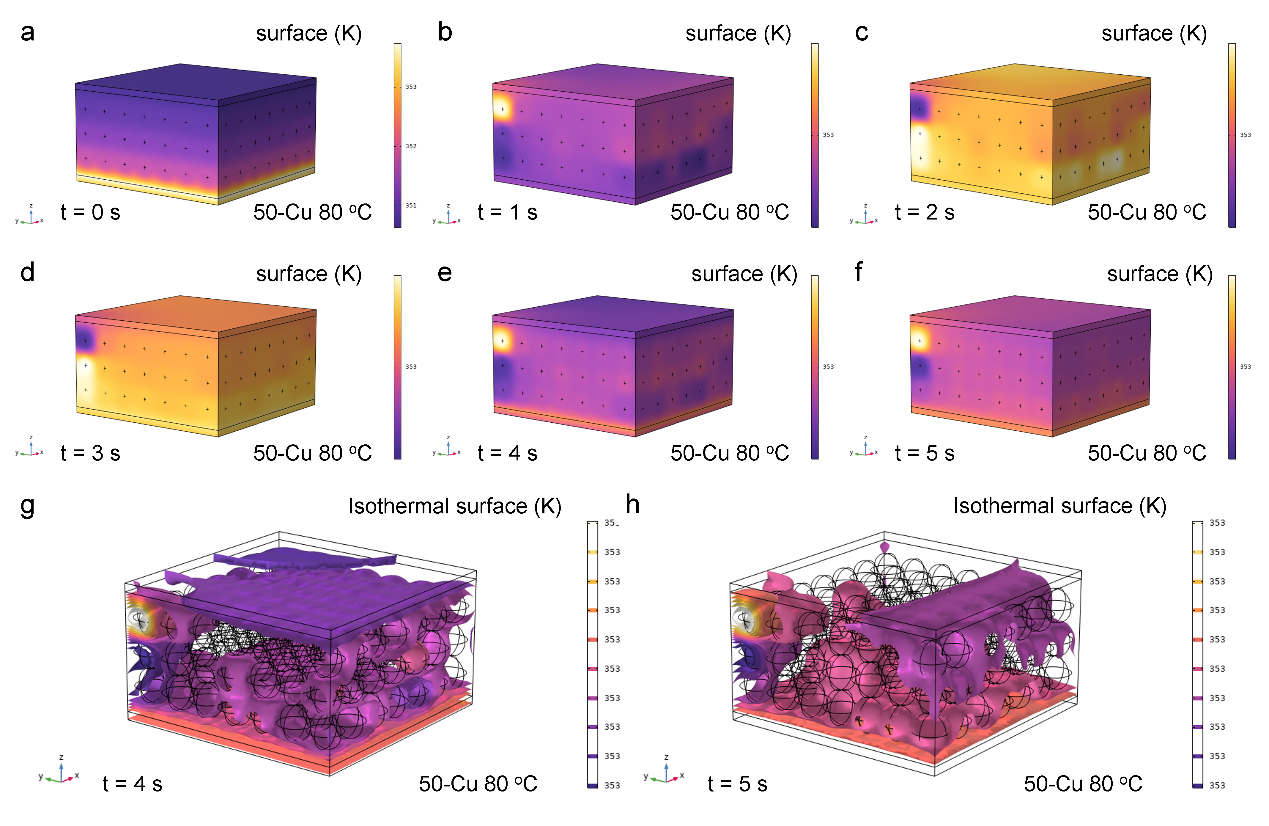
**Fig. S25.** Simulation diagram of thermal field distribution performed in COMSOL Software for 50-Cu sample (a-h).


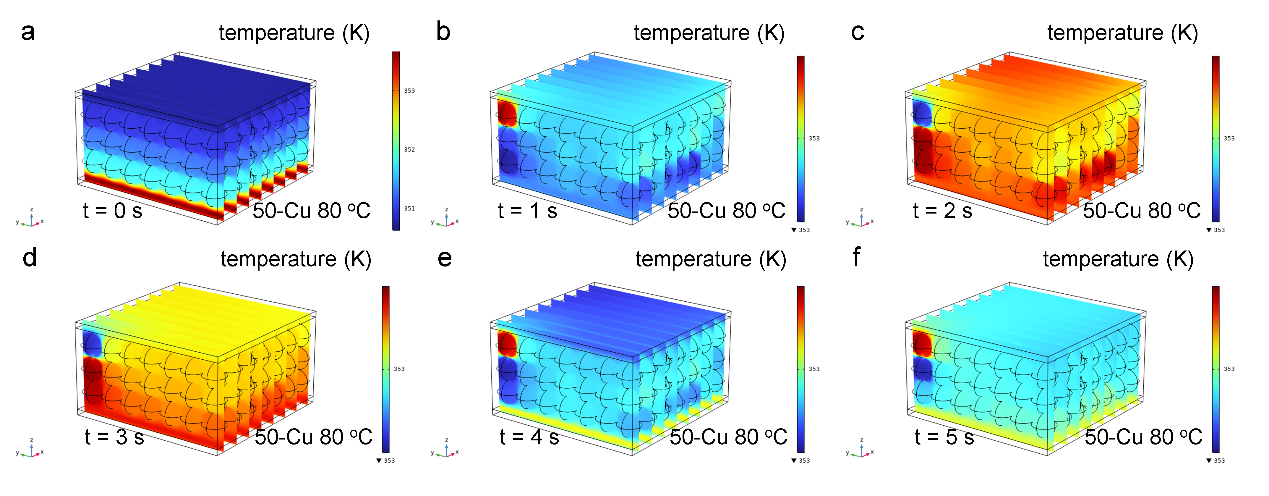
**Fig. S26.** Simulation diagram of thermal field distribution performed in COMSOL Software for 50-Cu sample (a-f).


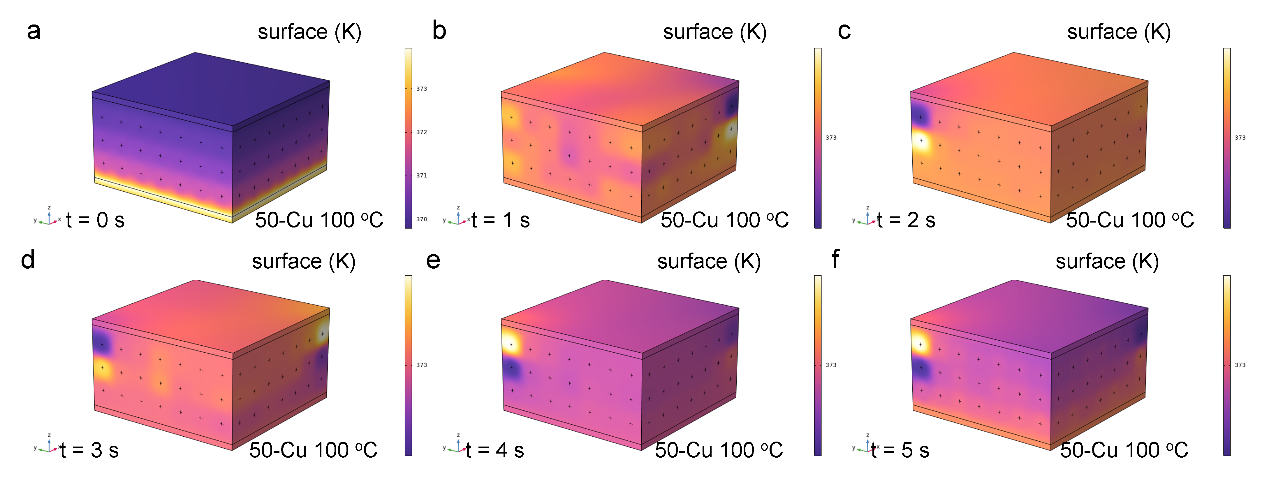
**Fig. S27.** Simulation diagram of thermal field distribution performed in COMSOL Software for 50-Cu sample (a-f).


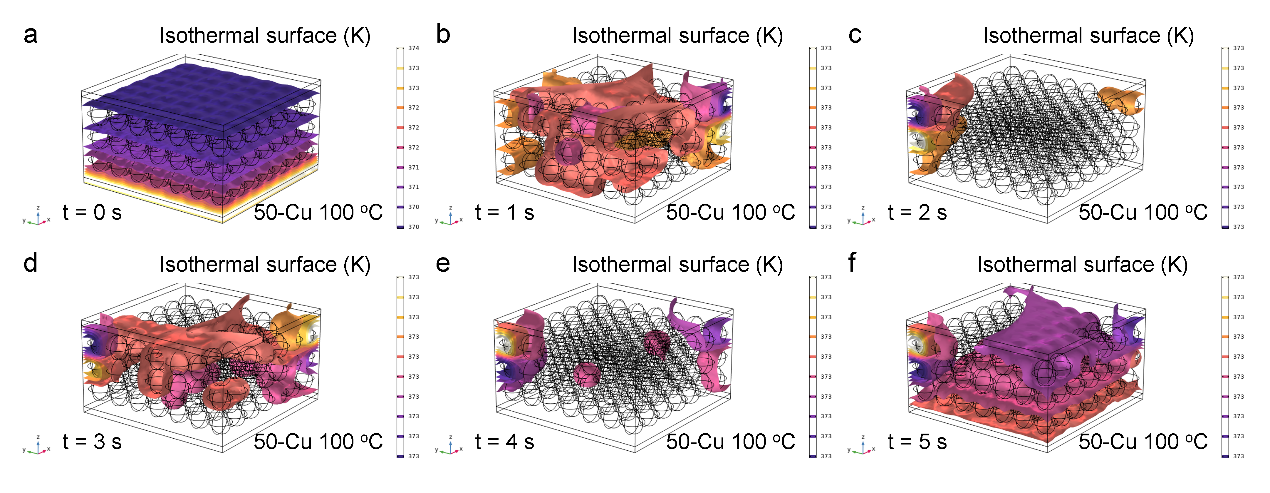
**Fig. S28.** Simulation diagram of thermal field distribution performed in COMSOL Software for 50-Cu sample (a-f).


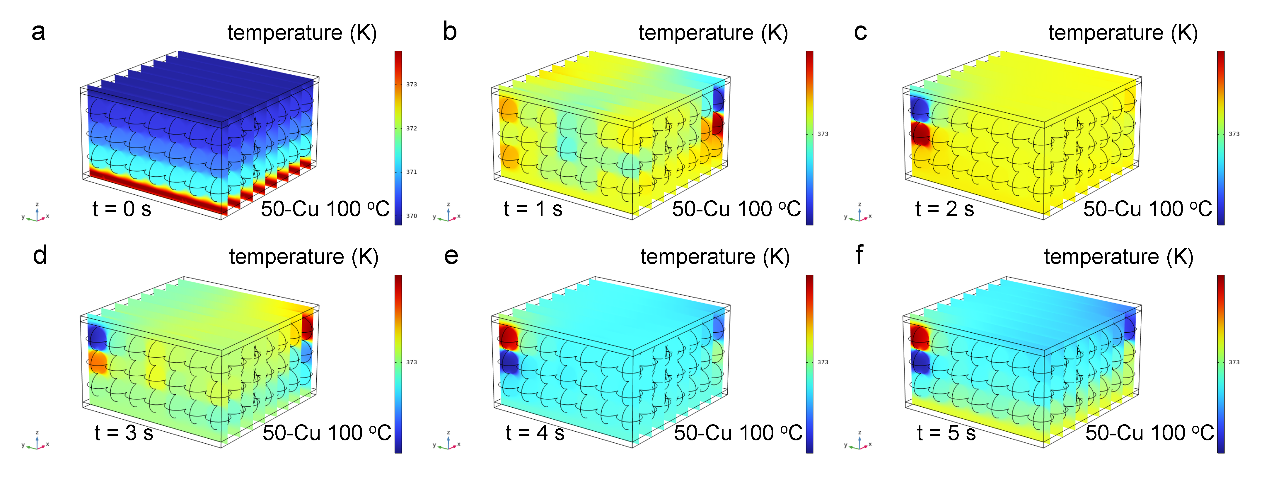
**Fig. S29.** Simulation diagram of thermal field distribution performed in COMSOL Software for 50-Cu sample (a-f).


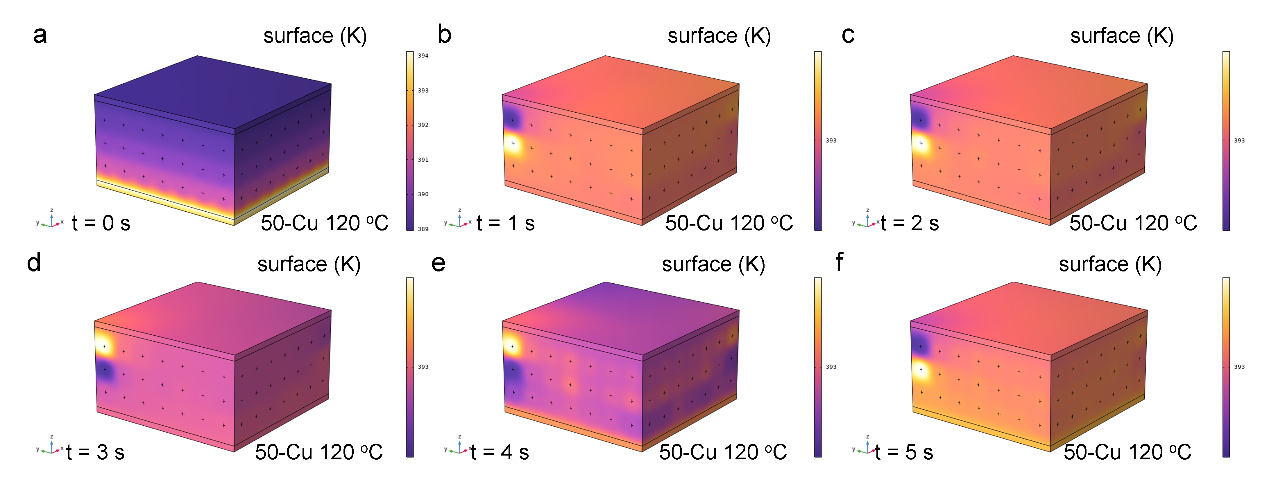
**Fig. S30.** Simulation diagram of thermal field distribution performed in COMSOL Software for 50-Cu sample (a-f).


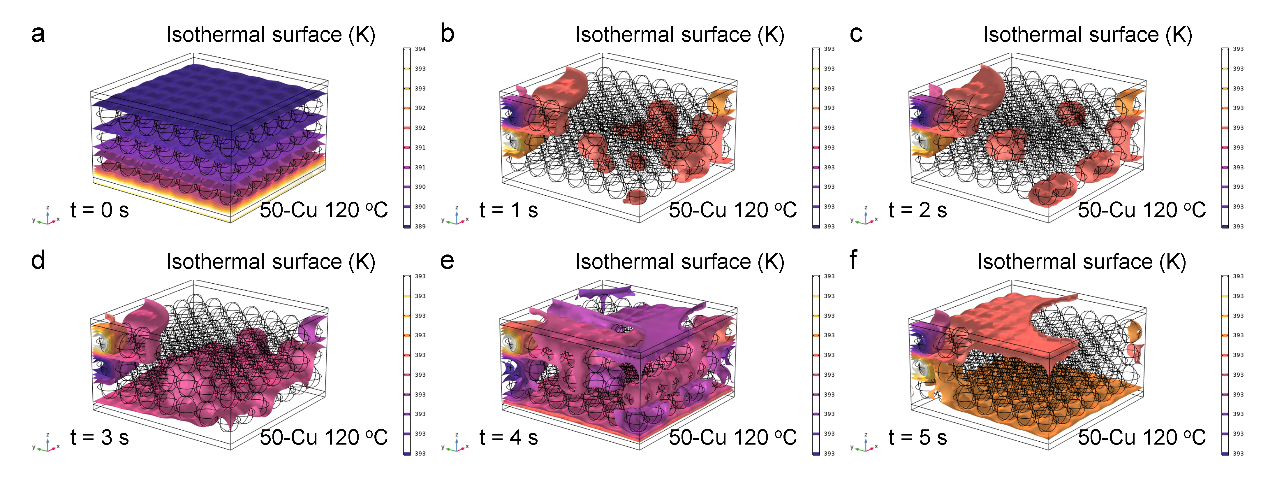
**Fig. S31.** Simulation diagram of thermal field distribution performed in COMSOL Software for 50-Cu sample (a-f).


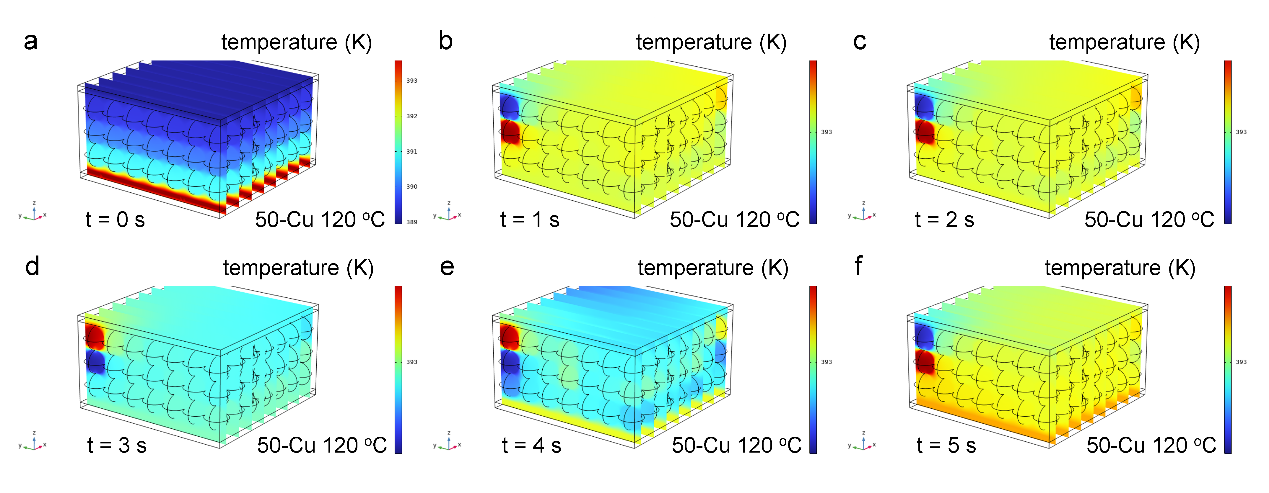
**Fig. S32.** Simulation diagram of thermal field distribution performed in COMSOL Software for 50-Cu sample (a-f).


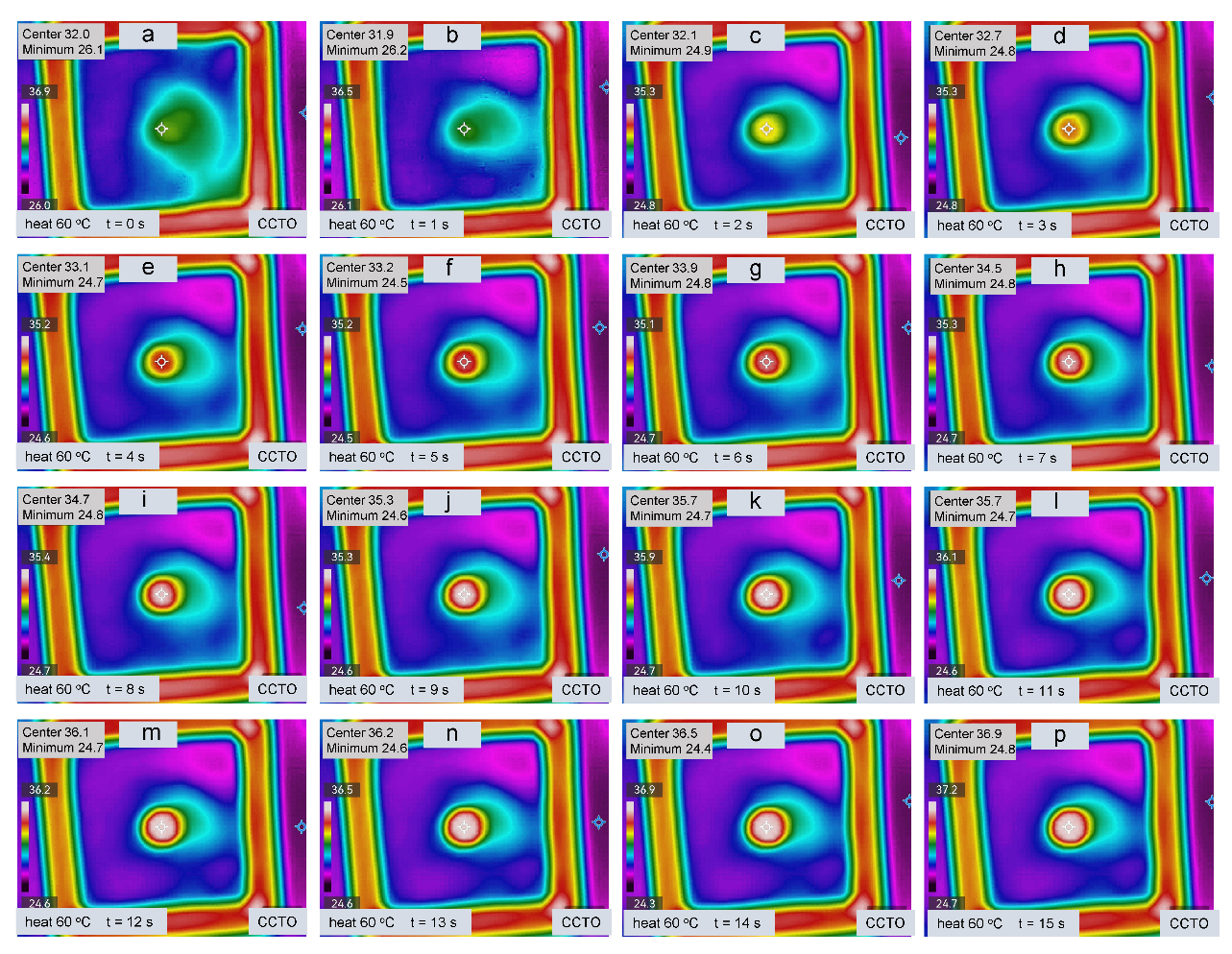
**Fig. S33.** Temperature pattern at heating temperature of 60 ^o^C for CCTO sample (a-p).


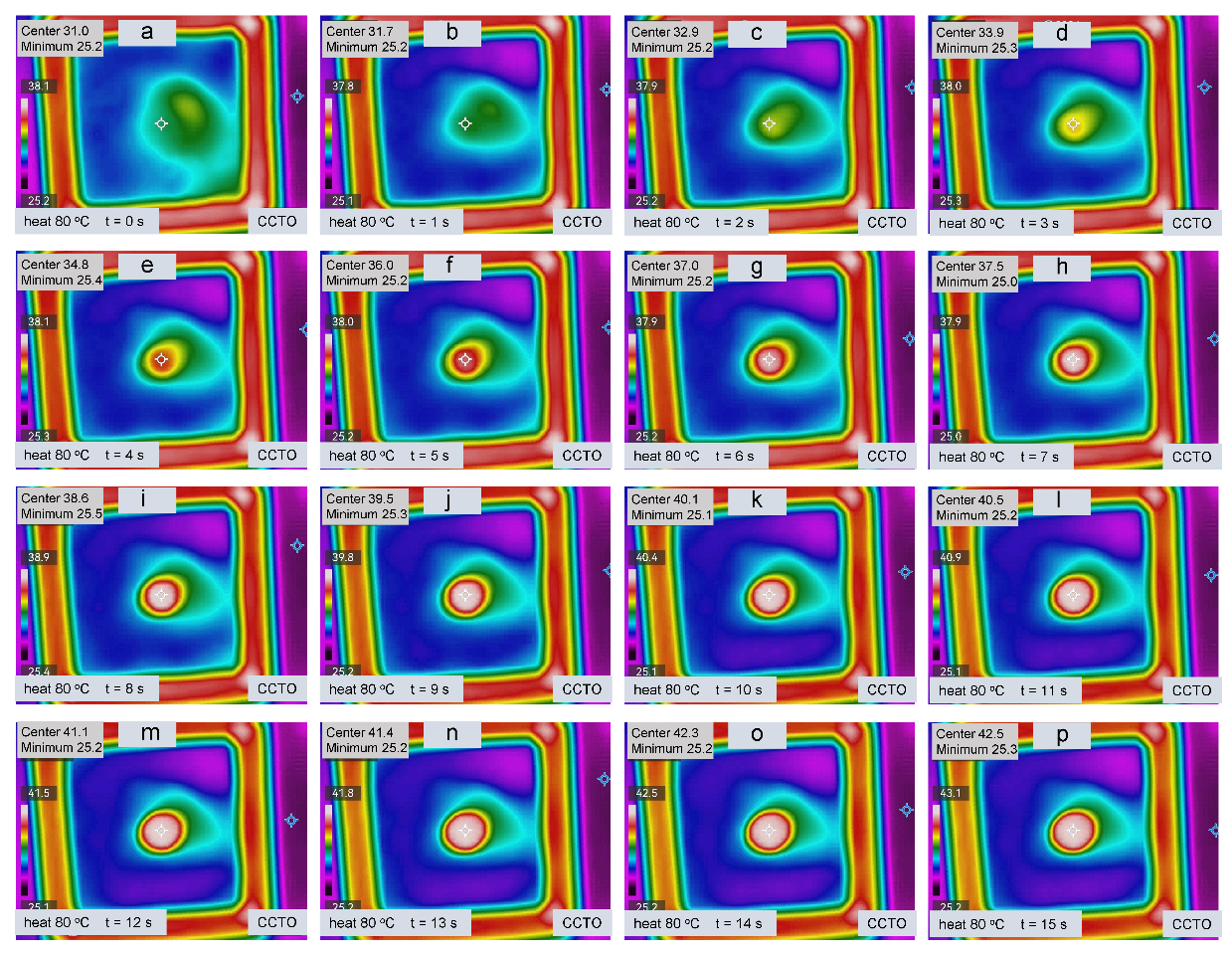
**Fig. S34.** Temperature pattern at heating temperature of 80 ^o^C for CCTO sample (a-p).


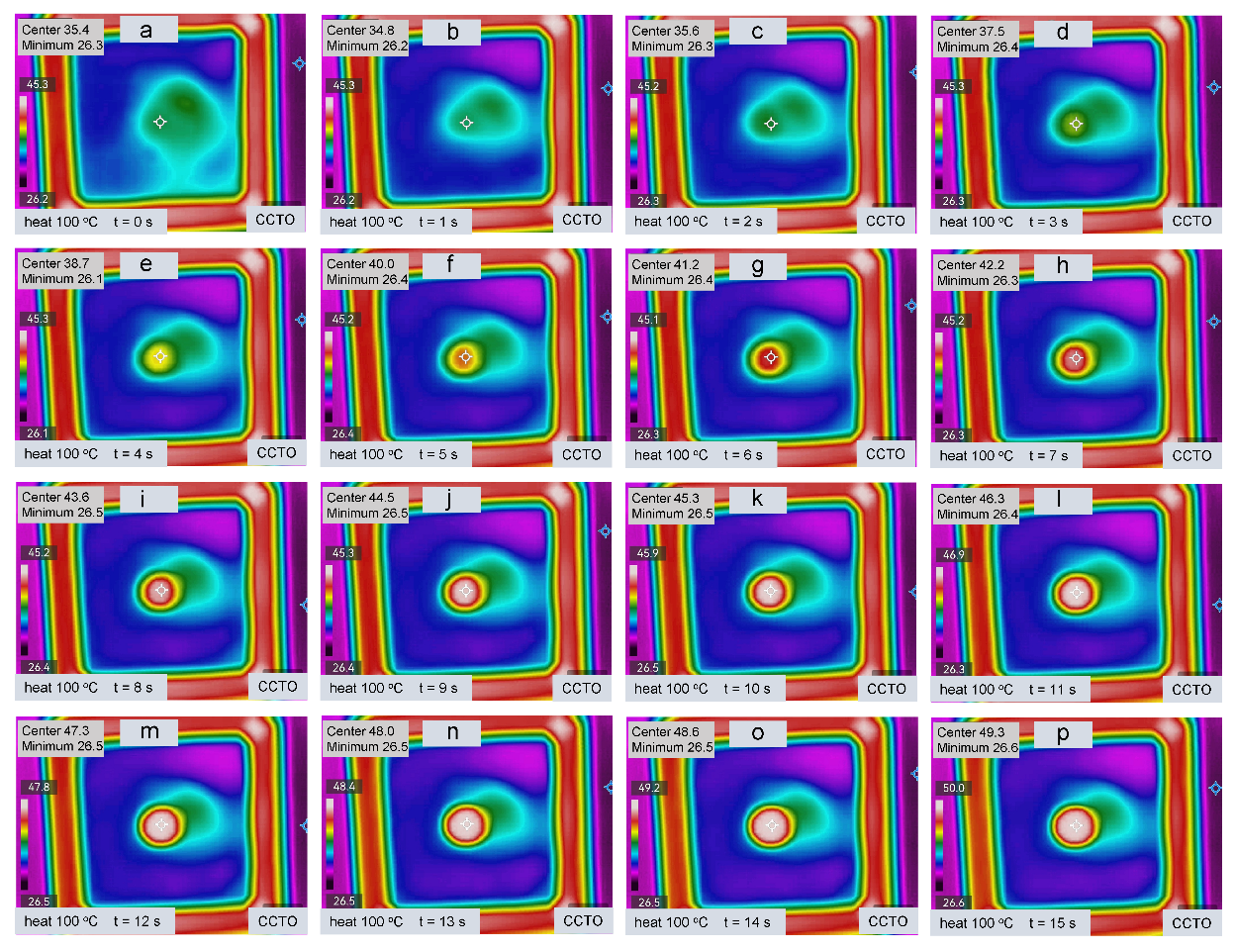
**Fig. S35.** Temperature pattern at heating temperature of 100 ^o^C for CCTO sample (a-p).


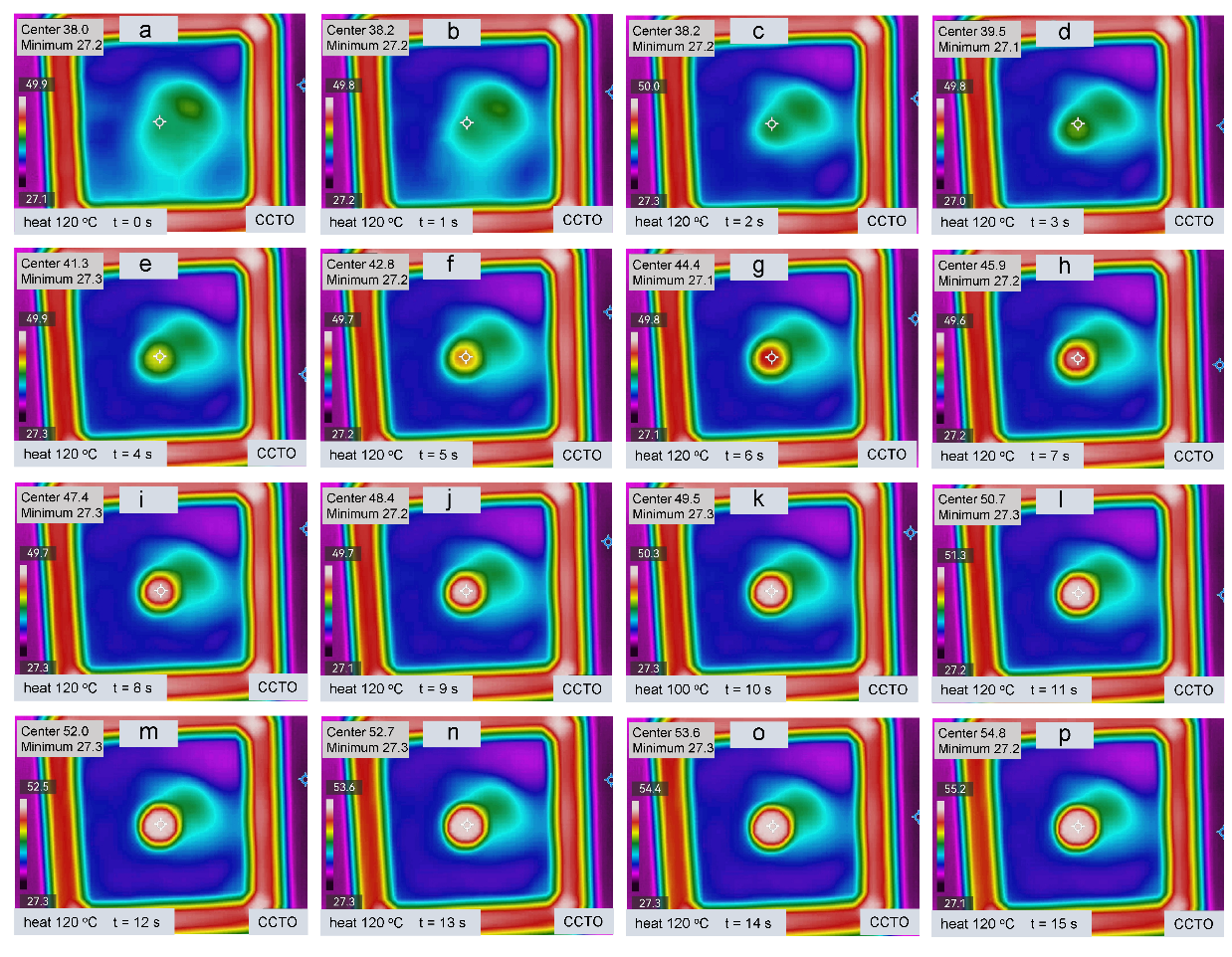
**Fig. S36.** Temperature pattern at heating temperature of 120 ^o^C for CCTO sample (a-p).


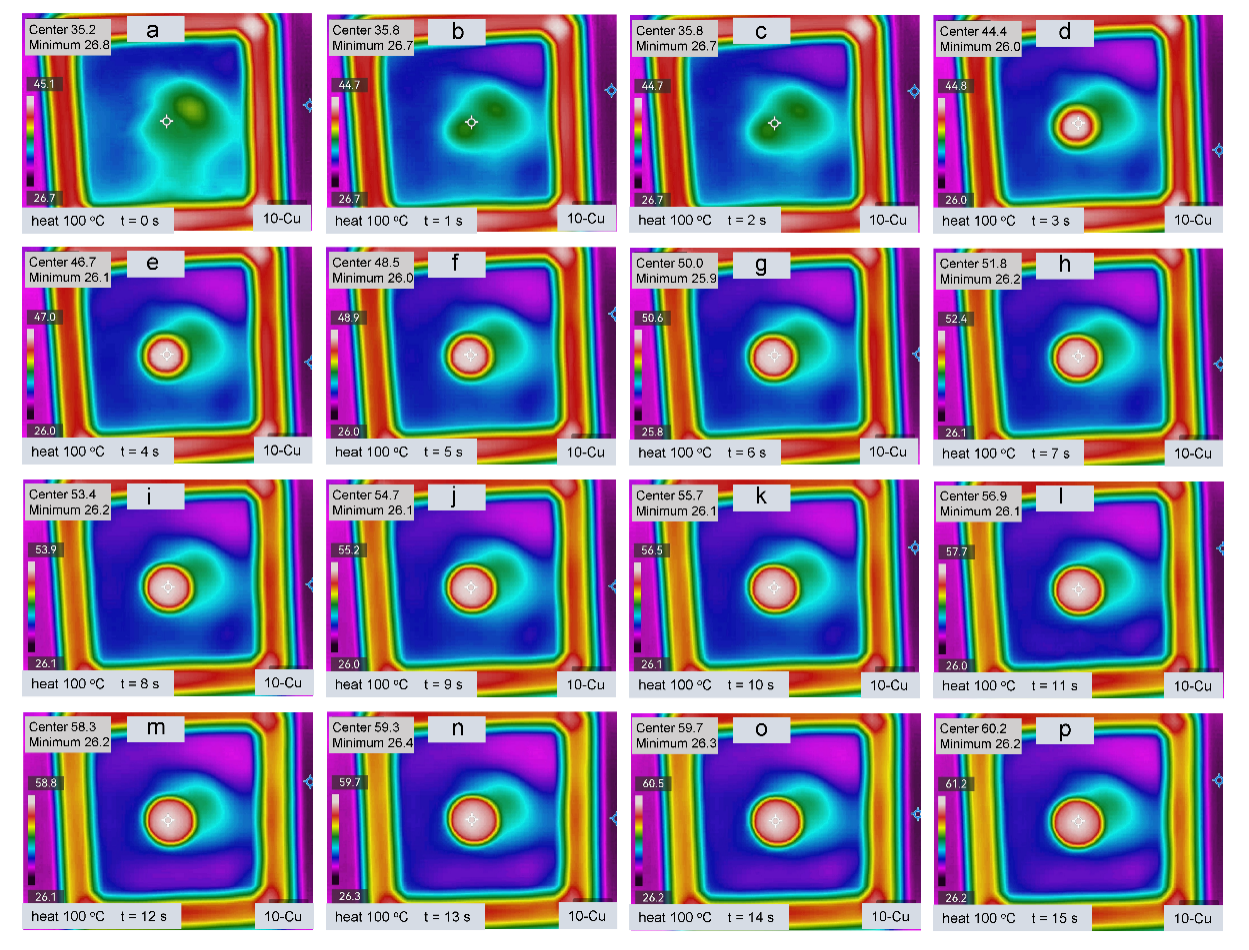
**Fig. S37.** Temperature pattern at heating temperature of 100 ^o^C for 10-Cu sample (a-p).


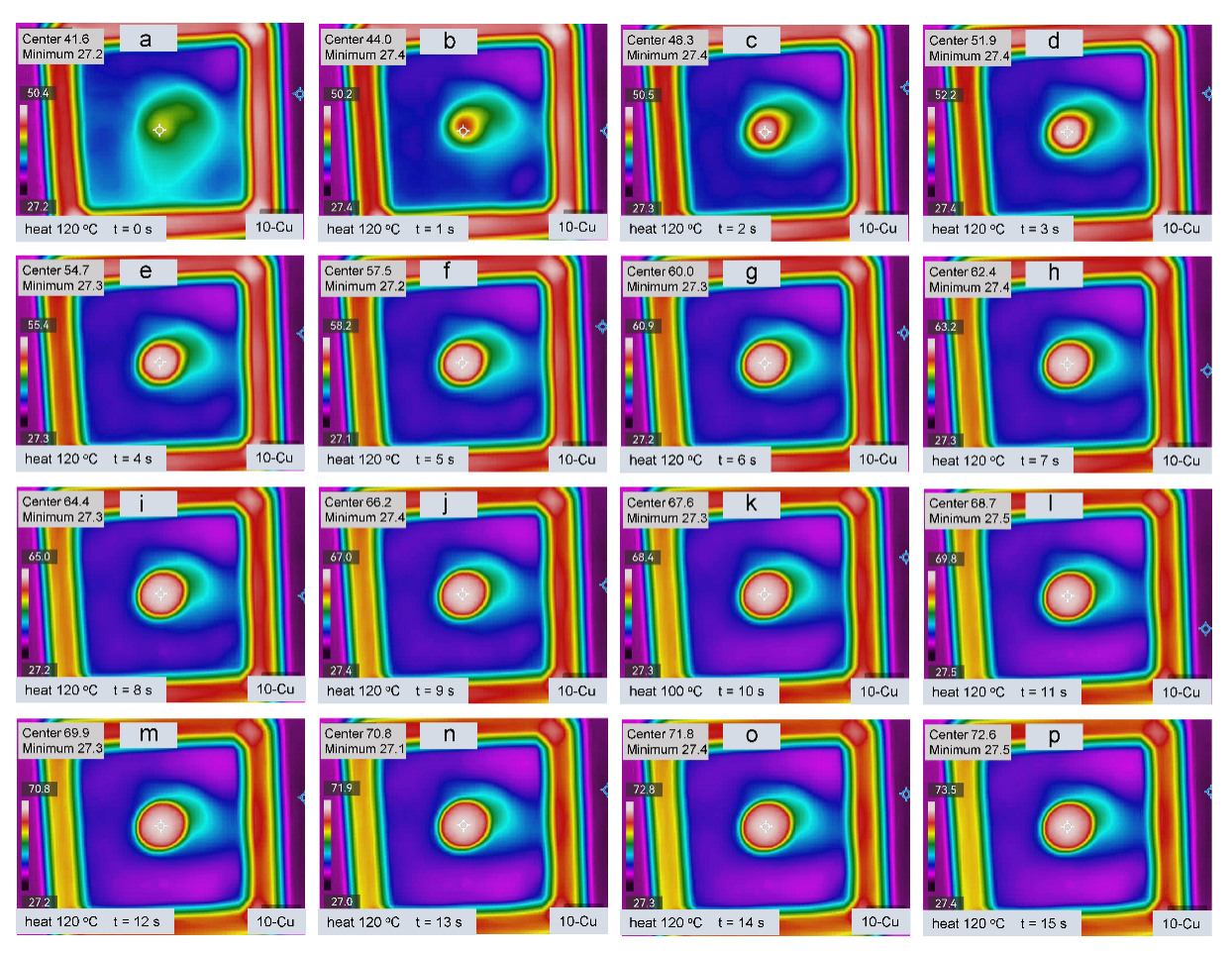
**Fig. S38.** Temperature pattern at heating temperature of 120 ^o^C for 10-Cu sample (a-p).


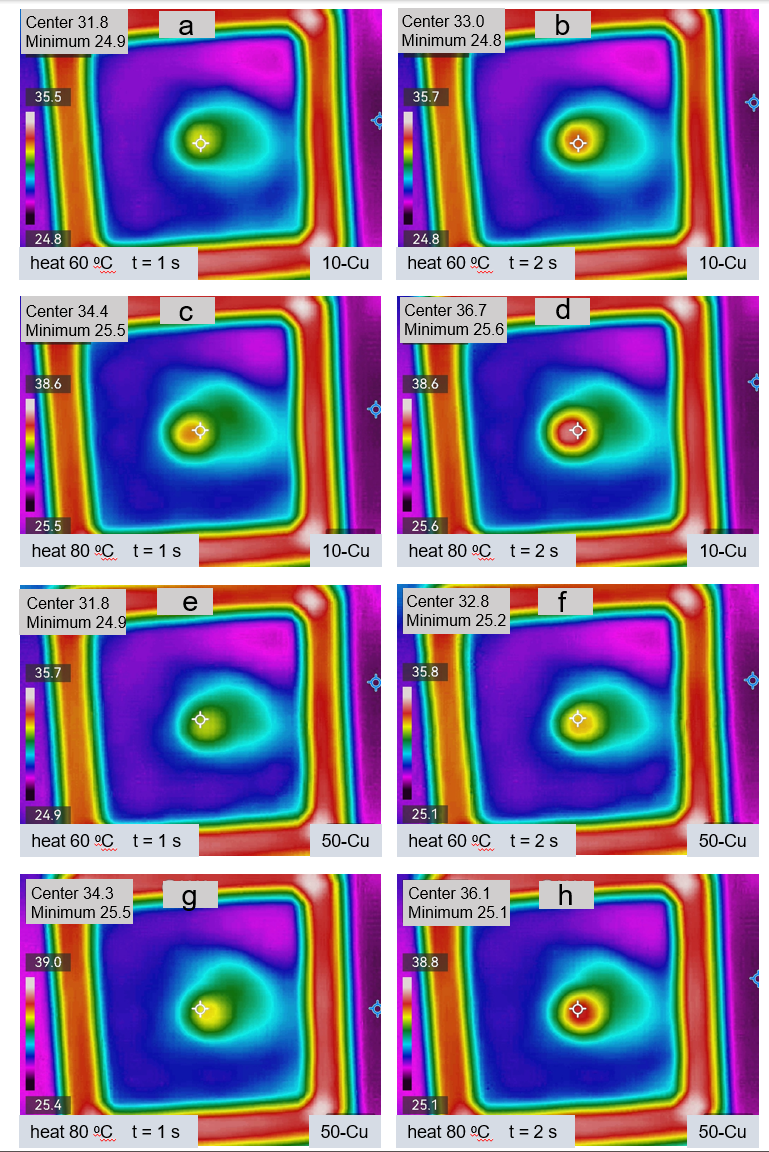


**Fig. S39.** Temperature pattern at heating temperature of 60 ^o^C /80 ^o^C for 10-Cu/50-Cu sample (a-h).


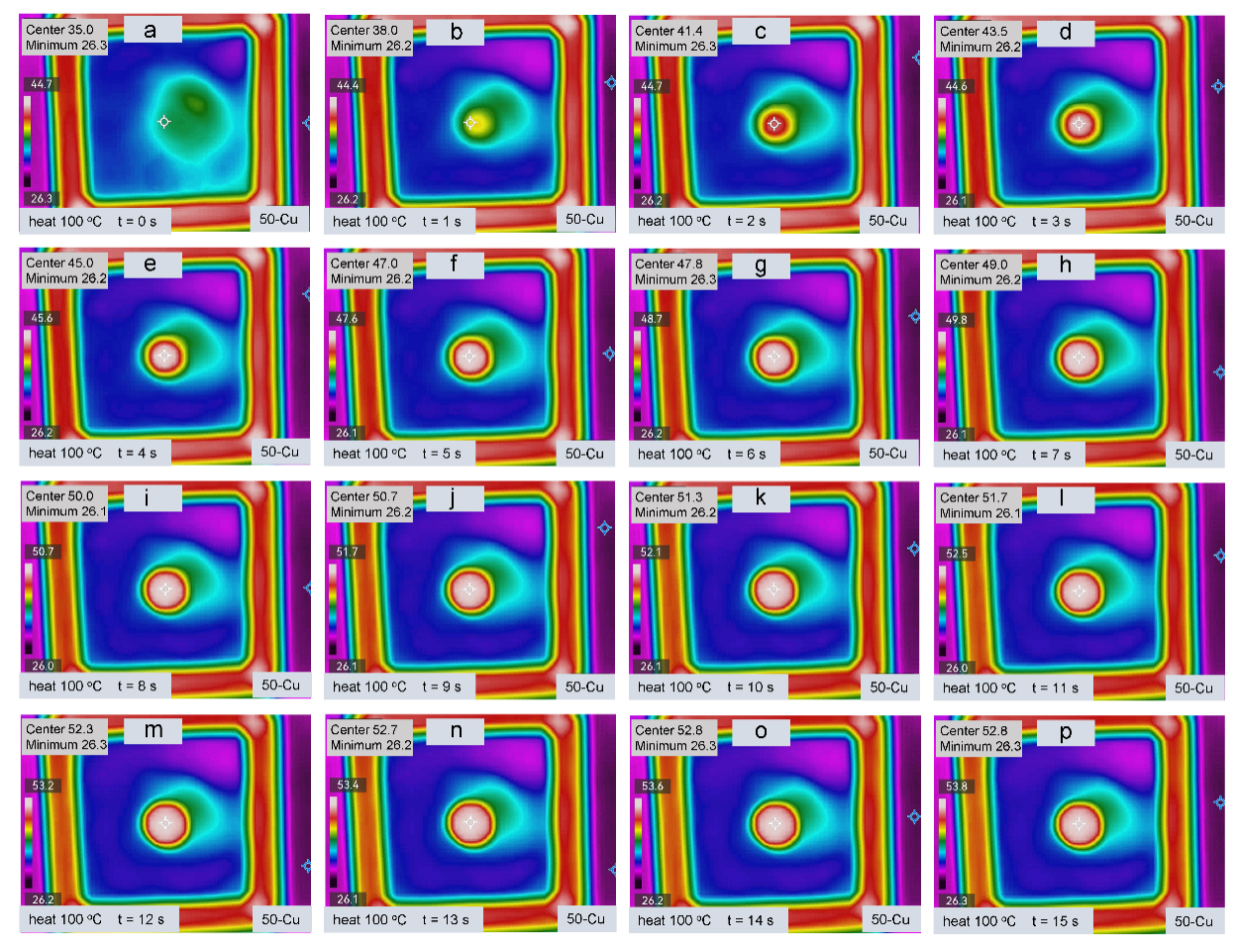
**Fig. S40.** Temperature pattern at heating temperature of 100 ^o^C for 50-Cu sample (a-p).


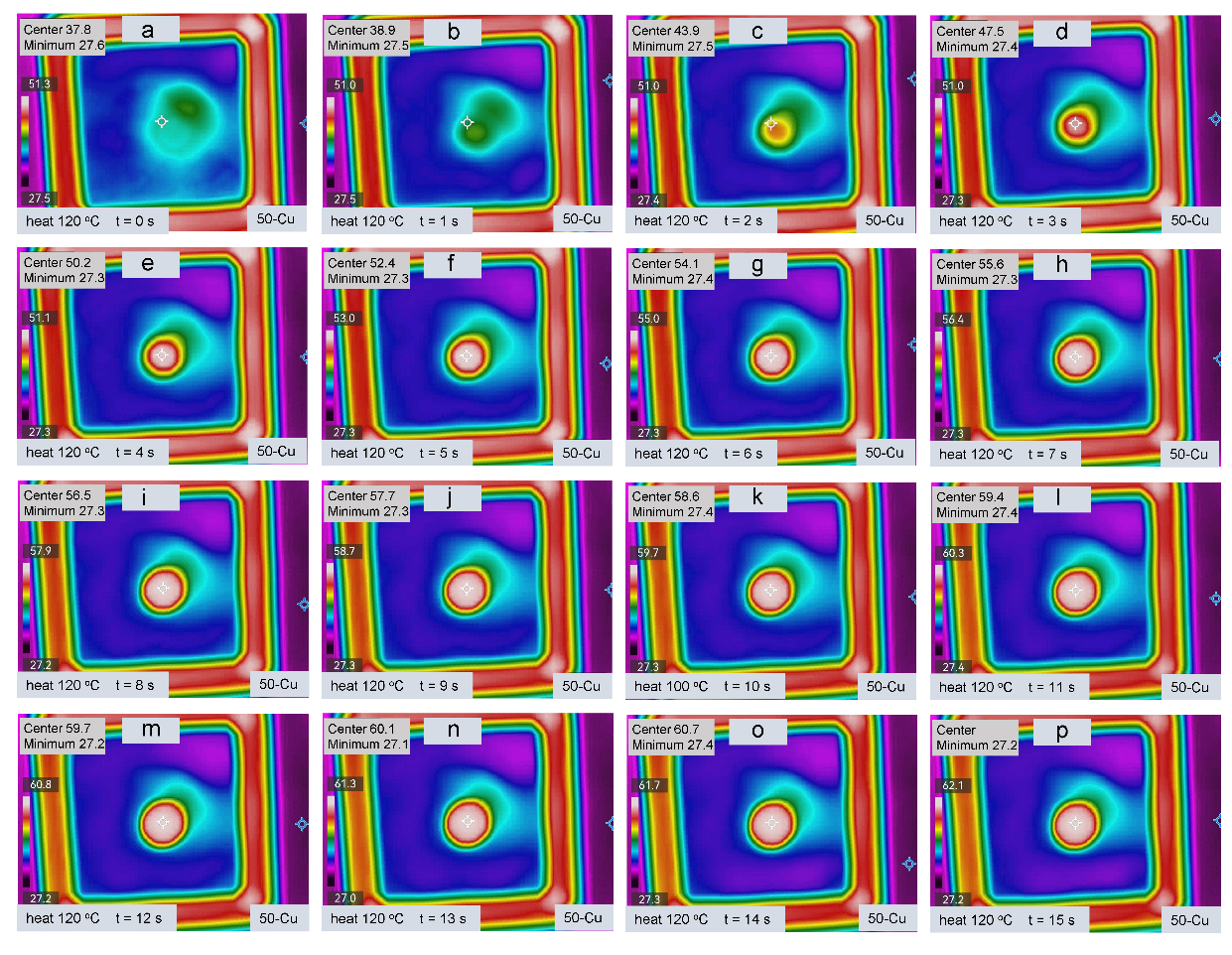
**FFig. S41.** Temperature pattern at heating temperature of 120 ^o^C for 50-Cu sample (a-p).
